# Author Correction: Direct binding of CEP85 to STIL ensures robust PLK4 activation and efficient centriole assembly

**DOI:** 10.1038/s41467-026-79190-5

**Published:** 2026-11-13

**Authors:** Yi Liu, Gagan D. Gupta, Deepak D. Barnabas, Fikret G. Agircan, Shahid Mehmood, Di Wu, Etienne Coyaud, Christopher M. Johnson, Stephen H. McLaughlin, Antonina Andreeva, Stefan M. V. Freund, Carol V. Robinson, Sally W. T. Cheung, Brian Raught, Laurence Pelletier, Mark van Breugel

**Affiliations:** 1 Lunenfeld-Tanenbaum Research Institute, Sinai Health System, 600 University Avenue, Toronto, ON M5G 1X5 Canada; 2 Department of Molecular Genetics, University of Toronto, Toronto, ON M5S 1A8 Canada; 3 Medical Research Council – Laboratory of Molecular Biology, Francis Crick Avenue, Cambridge, CB2 0QH UK; 4 Department of Chemistry, University of Oxford, Oxford, UK; 5 Princess Margaret Cancer Centre, University Health Network, 101 College Street, Toronto, ON M5G 1L7 Canada; 6 Department of Medical Biophysics, University of Toronto, Toronto, ON M5G 1L7 Canada

Correction to: *Nature Communications* https://doi.org/10.1038/s41467-018-04122-x, published online 30 April 2018

In the version of this article initially published, due to an error during figure assembly, the cyclin A blot presented in Fig. 2d was a duplication of the α-tubulin blot shown in Fig. 2l. Due to the age of the article, the figure cannot be updated directly. A revised Fig. 2 and uncropped western blots (revised Supplementary Fig. 11) are available as Supplementary Information accompanying this article. This notice serves to correct the article.

**Correct Fig. 2**

**Figure:**
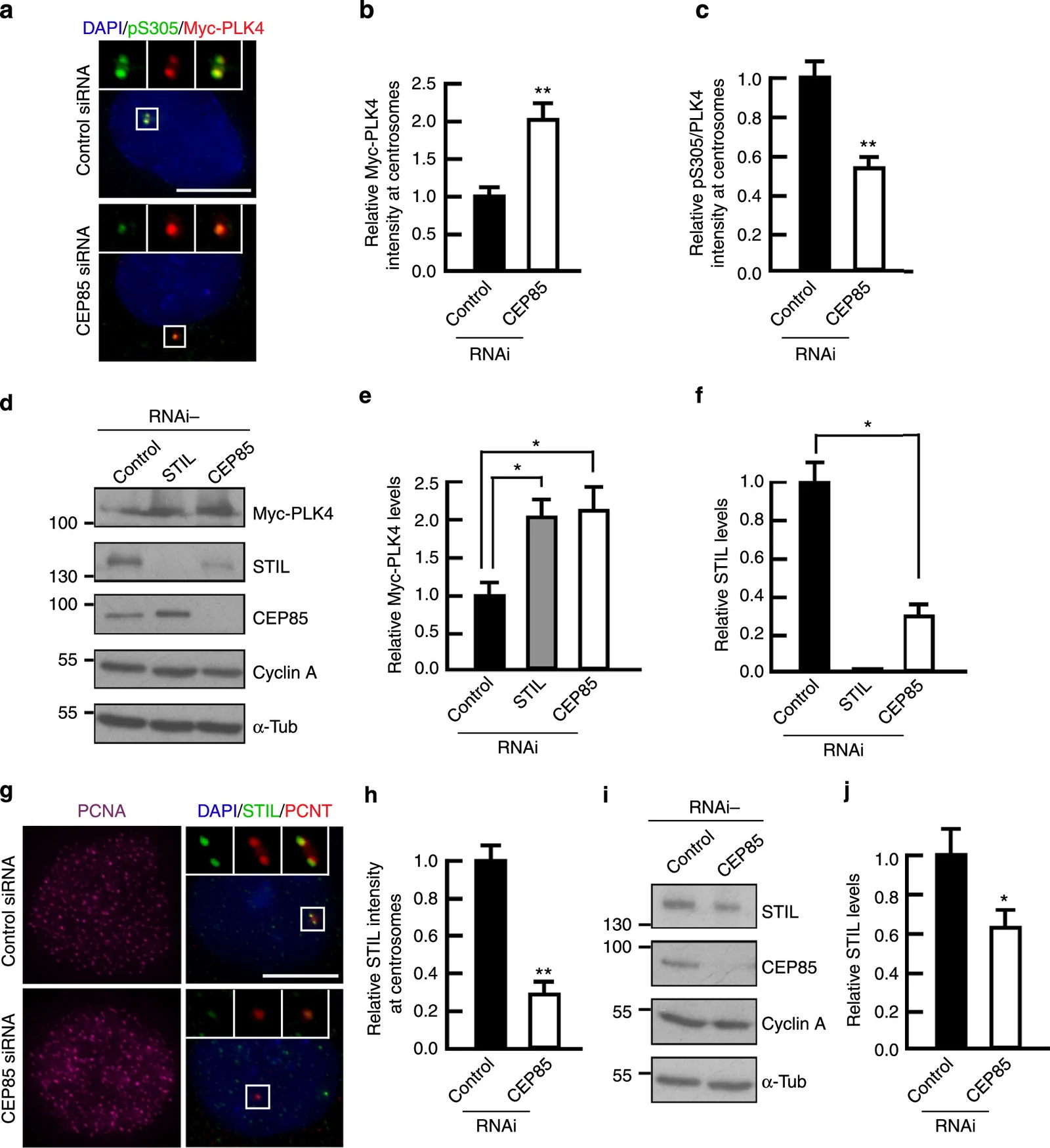

**Incorrect Fig. 2**

**Figure:**
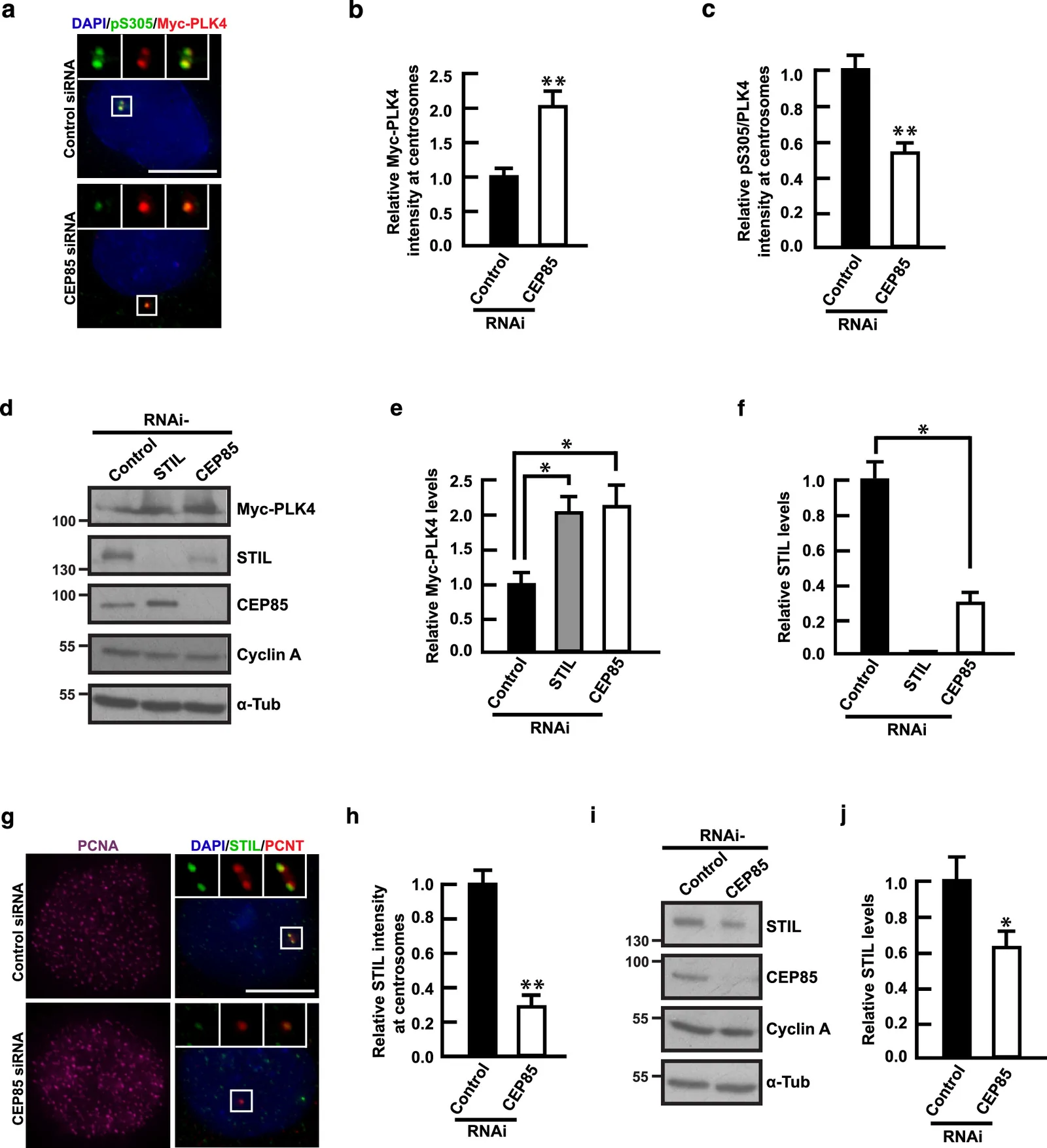

## Supplementary information

Corrected Fig. 2, Supplementary Fig. 11

