# Supplementary material for: Direct binding of CEP85 to STIL ensures robust PLK4 activation and efficient centriole assembly
Source: Nat Commun. 2018 Apr 30;9:1731. doi: 10.1038/s41467-018-04122-x (PMC5928214; doi:10.1038/s41467-018-04122-x)
Supplement: Supplementary file 1 — Supplementary Information [file 41467_2018_4122_MOESM1_ESM.pdf]

Supplementary Information

Direct binding of CEP85 to STIL ensures robust PLK4 activation and efficient centriole assembly

Liu et al.

Supplementary Figure 1

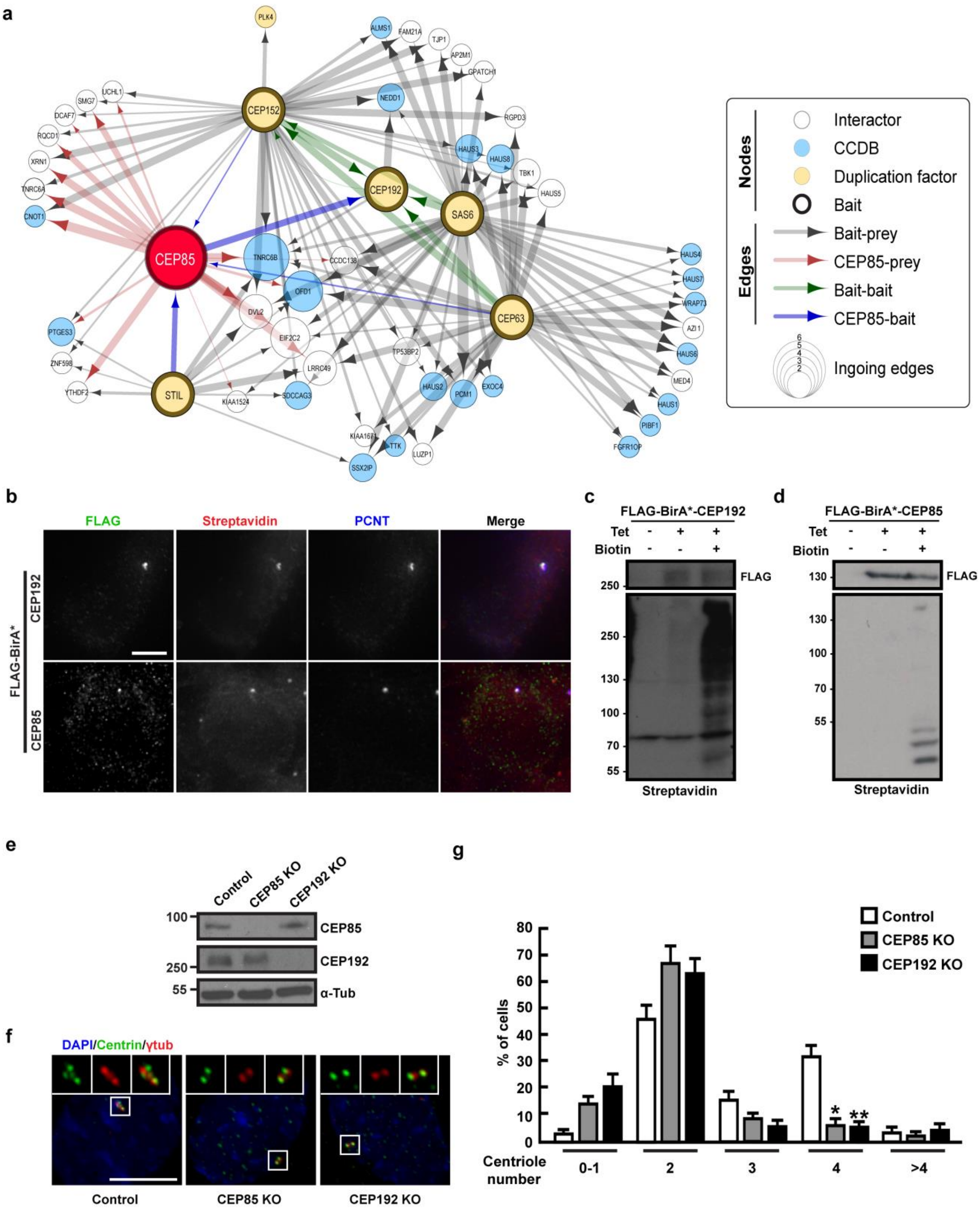

Supplementary Figure 1. Proximity interactions with centriolar components and effect of CEP85 KO on centriole assembly. (a). Spring-embedded BioID network of centriole duplication factors. Shown are high-confidence interactors detected with two or more bait proteins. Bait, interactor and edges are colour-coded as indicated in the legend. Edge thickness is proportional to total peptide counts (see Supplementary Dataset 1). **(b).** IF analysis of HEK293 cells expressing FLAG-BirA*-CEP192 and CEP85 labelled with anti-PCNT and anti-FLAG antibodies, and fluorophore coupled Streptavidin. Tet and Biotin were added to induce the expression of the fusion proteins and trigger the BirA* biotin ligase. Scale bar 5 μm. **(c, d).** Western blot indicates the expression of FLAG-BirA*-CEP192 and CEP85 and detection of biotinylated proteins using Streptavidin as shown in b. **(e, f).** Western blot and IF analysis of lentivirus control (vector), lentivirus CRISPR CEP85 or CEP192 guide RNAs treated RPE-1 cells, labelled with the indicated antibodies. Selected images showing Centrin and γ-tubulin labelling. Scale bar 10 μm, white boxes indicate the magnified region. **(g).** The graph shows the percentage of cells with the indicated centriole numbers (n = 100/experiment, three independent experiments). Two-tailed t-test was performed for all p-values, all error bars represent S.D., and asterisks for p-values are $**p<0.01$ and $*p<0.05$.

Supplementary Figure 2

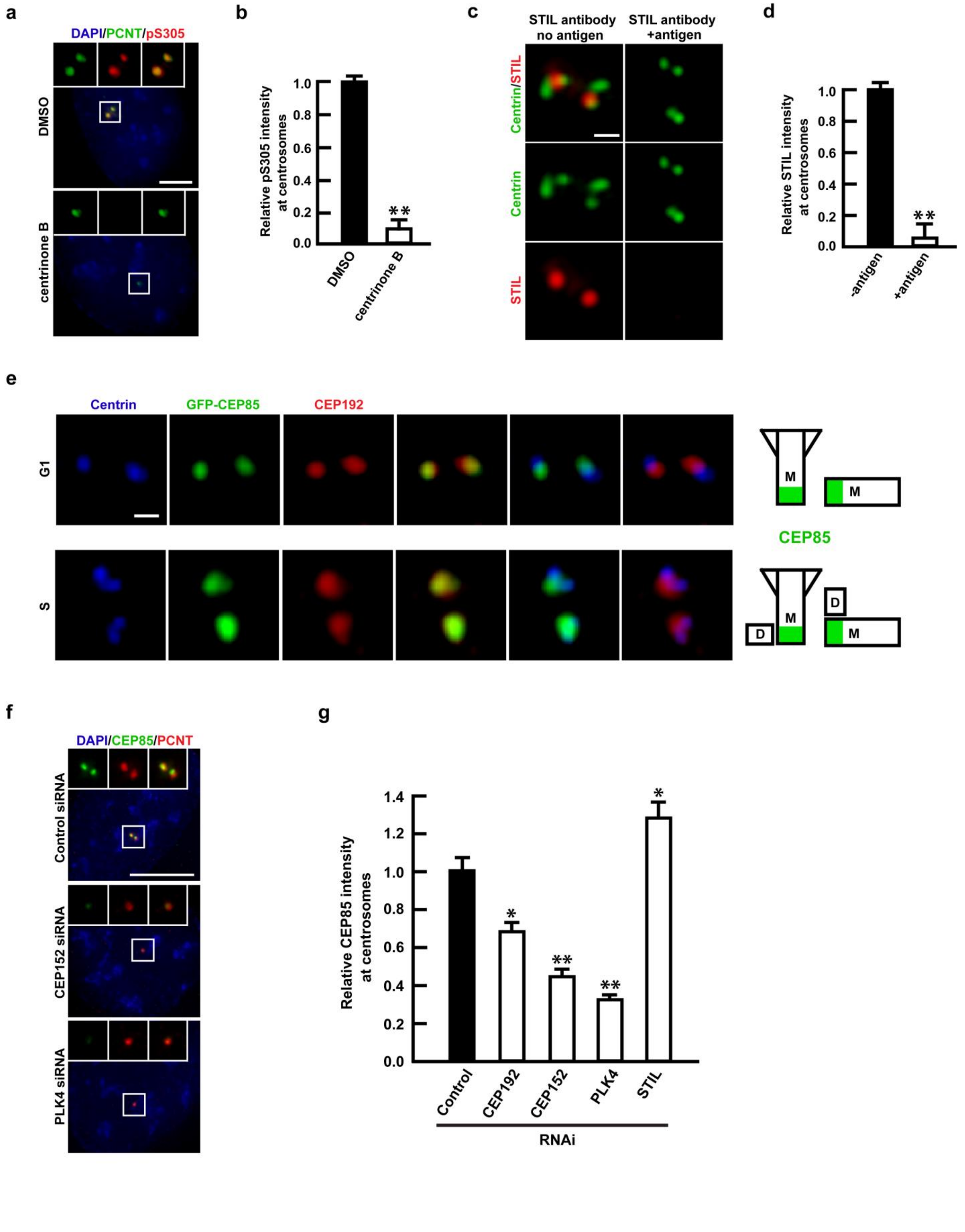

Supplementary Figure 2. Specificity of PLK4 pS305 and STIL antibodies, colocalization between CEP85 and CEP192, and requirements of CEP85 localization to centrosomes. (a, b). The PLK4-induced centriole overduplication assay was performed as described in Materials and Methods. DMSO and centrinone B (2500 nM) were added for 24 h berfore fixation. Cells were labelled with DAPI and the indicated antibodies. Scale bar 5 μm, white boxes indicate the magnified region. **(b).** The graph shows the relative levels of PLK4 pS305 at centrosomes (n = 100/experiment, three independent experiments). **(c, d).** The anti-STIL antibody was pre-absorbed with the corresponding antigen before the IF experiments in U-2 OS cells. Selected images indicating STIL and Centrin labelling. Scale bar 0.5 μm. **(d).** Quantification showing the relative levels of STIL at centrosomes (n = 100/experiment, three independent experiments). **(e).** Determining the centriolar localization of CEP85. Cells were transfected with the GFP-CEP85 construct and stained with the indicated antibodies. Scale bar 0.5 μm. Schematic representation of CEP85 (green) localization on the proximal end of mother centrioles. **(f).** Factors required for the recruitment of CEP85 to centrioles. Centriolar levels of CEP85 during centriole duplication were assessed using the PLK4-induced centriole overduplication assays (See the Materials and Methods). Selected images of CEP85 and PCNT labelling are shown. Scale bar 10 μm, white boxes indicates the magnified region. **(g).** The graph indicates the relative intensity of CEP85 at centrosomes (n = 100/experiment, three independent experiments). Two-tailed t-test was performed for all p-values, all error bars represent S.D., and asterisks for p-values are $^{**}p<0.01$ and $^{*}p<0.05$.

Supplementary Figure 3

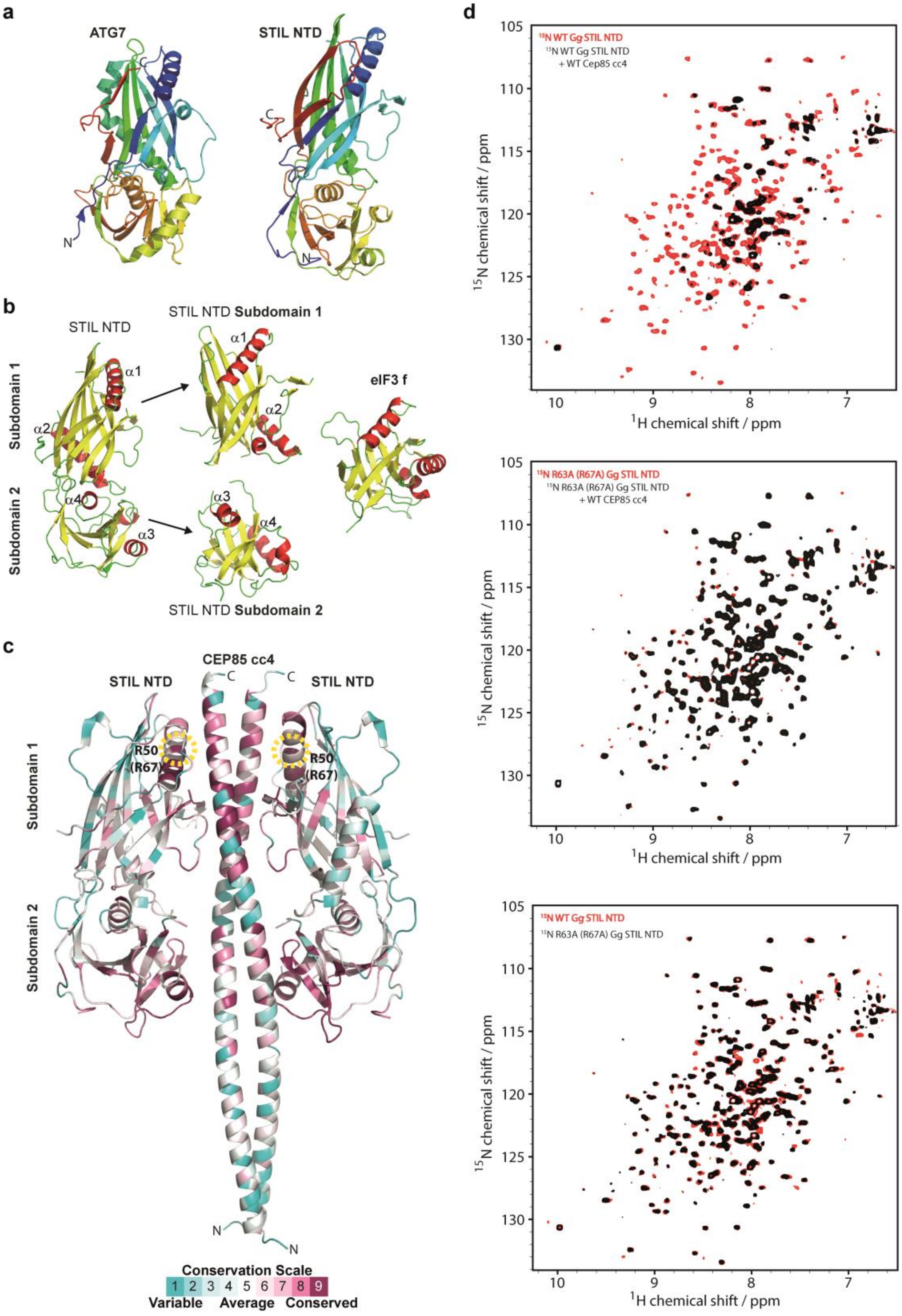

Supplementary Figure 3. Structural analysis of STIL NTD and its binding to CEP85 cc4. **(a).** Side-by-side comparison of *Trichoplax adhaerens* STIL NTD and the N-terminal domain of ATG7[1] (PDB 3T7G, ribbon-presentation, rainbow-coloured from N- to C-terminus). **(b).** Comparison of the two subdomains of *T.adhaerens* STIL NTD and eIF3f[2] (PDB 5a5t, ribbon-presentation, α-helices, red; β-sheets, yellow; loops, green). The structural core of these domains has a comparable topology comprising a six-stranded β-barrel capped at the top and on one side with helices. The resemblance between STIL NTD subdomain 1 and eIF3f extends to the crossover hairpin connecting strands 4 and 7. **(c).** Putative model of the *T.adhaerens* STIL NTD : human CEP85 cc4 complex with a fully extended CEP85 cc4 (ribbon-presentation coloured by CONSURF evolutionary conservation score from unconserved (cyan) to highly conserved (burgundy)). In our experimental model of the *T.adhaerens* STIL NTD: human CEP85 cc4 complex, the N-terminal half of CEP85 cc4 was not visible probably due to partial unfolding of this region in the crystal (compare with Figure 5C). To model the complex with an intact CEP85 cc4 dimer, we replaced the CEP85 cc4 rump structure with our high-resolution structure of CEP85 cc4. Note that the two conserved patches in STIL NTD subdomain 1 and 2 face towards CEP85 cc4 and that their putative contact regions in CEP85 cc4 are also conserved. **(d).** Overlay of ^{1}H,^{15}N BEST-TROSY NMR spectra of the wild-type and R63A *Gallus gallus* STIL NTD (R67A in human STIL NTD) in solution. Top panel: Addition of wild-type human CEP85 cc4 to ^{15}N wild-type chicken STIL NTD leads to a substantial loss of signals indicating the formation of a higher molecular weight complex. In contrast, adding wild-type human CEP85 cc4 to ^{15}N R63A chicken STIL NTD (middle panel) shows little effect, indicating the dominant role of the interface affected by this mutation. Bottom panel: The R63A mutant retains the overall structure of chicken STIL NTD and introduces only very minor (local) perturbations. Residue numbering is based on the corresponding STIL homologues used (equivalent residue numbers in human STIL are indicated in brackets).

Supplementary Figure 4

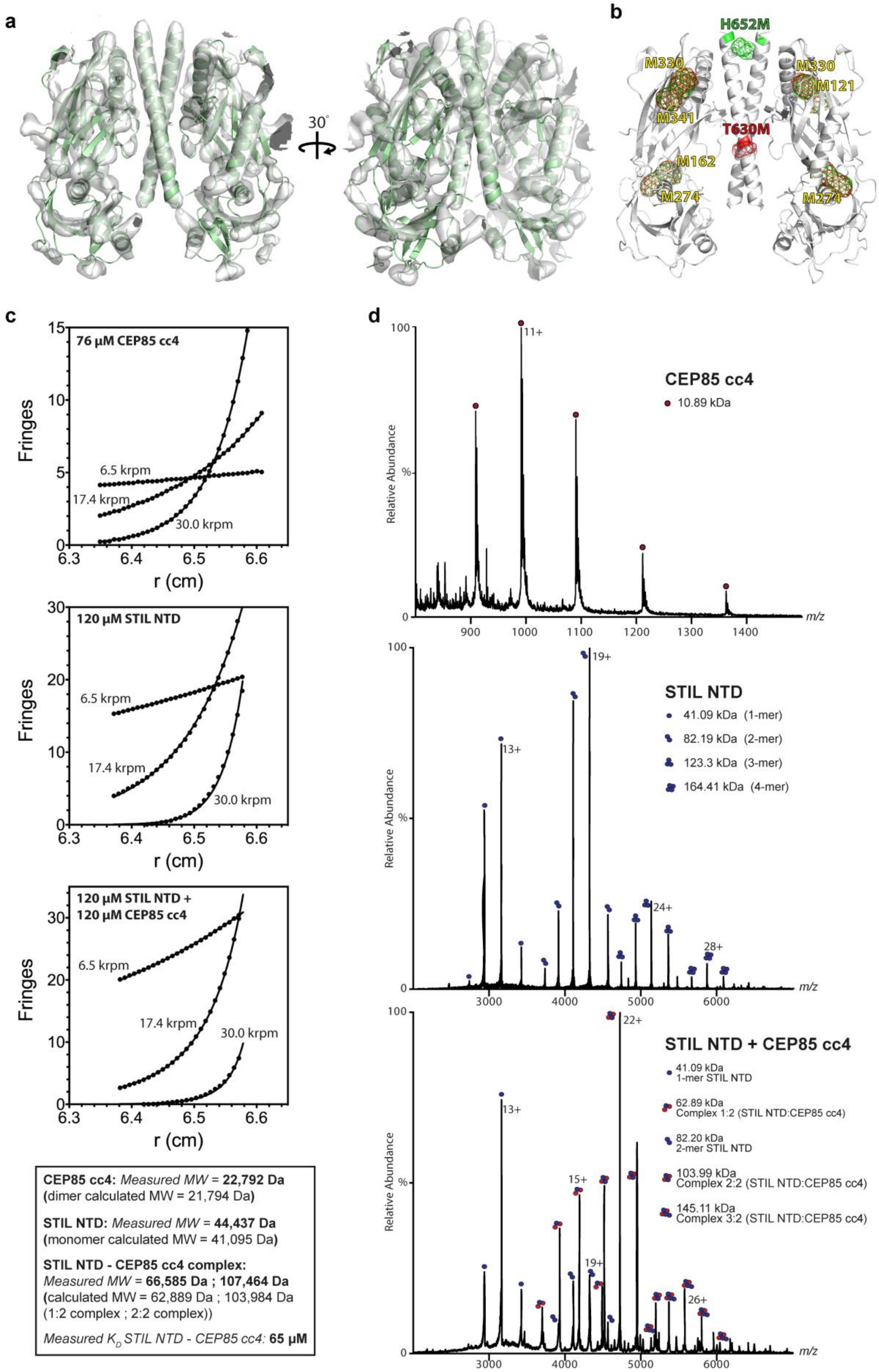

Supplementary Figure 4. Recombinant STIL NTD and CEP85 cc4 bind each other across species. (a). Experimental electron density map of the *Trichoplax adhaerens* STIL NTD : human CEP85 cc4 complex (as semi-transparent, grey isosurface, contour level σ=1.5), with the ribbon-presentation of the structure shown in green. **(b).** Ribbon-presentation of the *T.adhaerens* STIL NTD: human CEP85 cc4 complex (grey) together with an isomesh representation of the phased anomalous difference map (contour level σ=4.5) of the CEP85 cc4 T630M (red) and H652M (green) mutant of this complex. Maps show the selenium positions in the crystallized selenomethionine derivatives of the corresponding complexes. The selenomethionine Cα positions in the model are coloured in green and red in CEP85 cc4 and yellow in *T.adhaerens* STIL NTD. **(c).** Sedimentation equilibrium analysis of the molecular weights of human CEP85 cc4 and chicken STIL NTD alone and in complex in solution. Filled circles show the sedimentation equilibrium profiles at different speeds, solid lines display the fits of the data using either an average mass model or a heterodimeric association model in which two monomers of chicken STIL NTD interact with a dimer of human CEP85 cc4. Derived molecular weights and binding affinity are indicated. **(d).** Nanospray native mass spectra of recombinantly purified human CEP85 cc4 (top), chicken STIL NTD (middle) and chicken STIL NTD: human CEP85 cc4 (bottom) at 25 µM in solution. The measured molecular weights are indicated. Chicken STIL NTD and human CEP85 cc4 are labelled as violet and red circle, respectively. Mass spectra showed that chicken STIL NTD is present under the conditions of the experiment as monomer, dimer, trimer and tetramer, while human CEP85 cc4 was monomeric. The chicken STIL NTD: human CEP85 cc4 complex showed mainly formation of a heterotetramer with a stoichiometry of 2:2. Also observed are some populations of CEP85 cc4 dimer interacting with chicken STIL NTD monomer and trimer. The CEP85 cc4 monomer shows no interaction with any oligomeric form of chicken STIL NTD under the analysed protein concentrations. The tendency of STIL NTD to oligomerise was not observed in MALS (Supplementary Figure 5a) or AUC (Supplementary Figure 4c) experiments.

Supplementary Figure 5

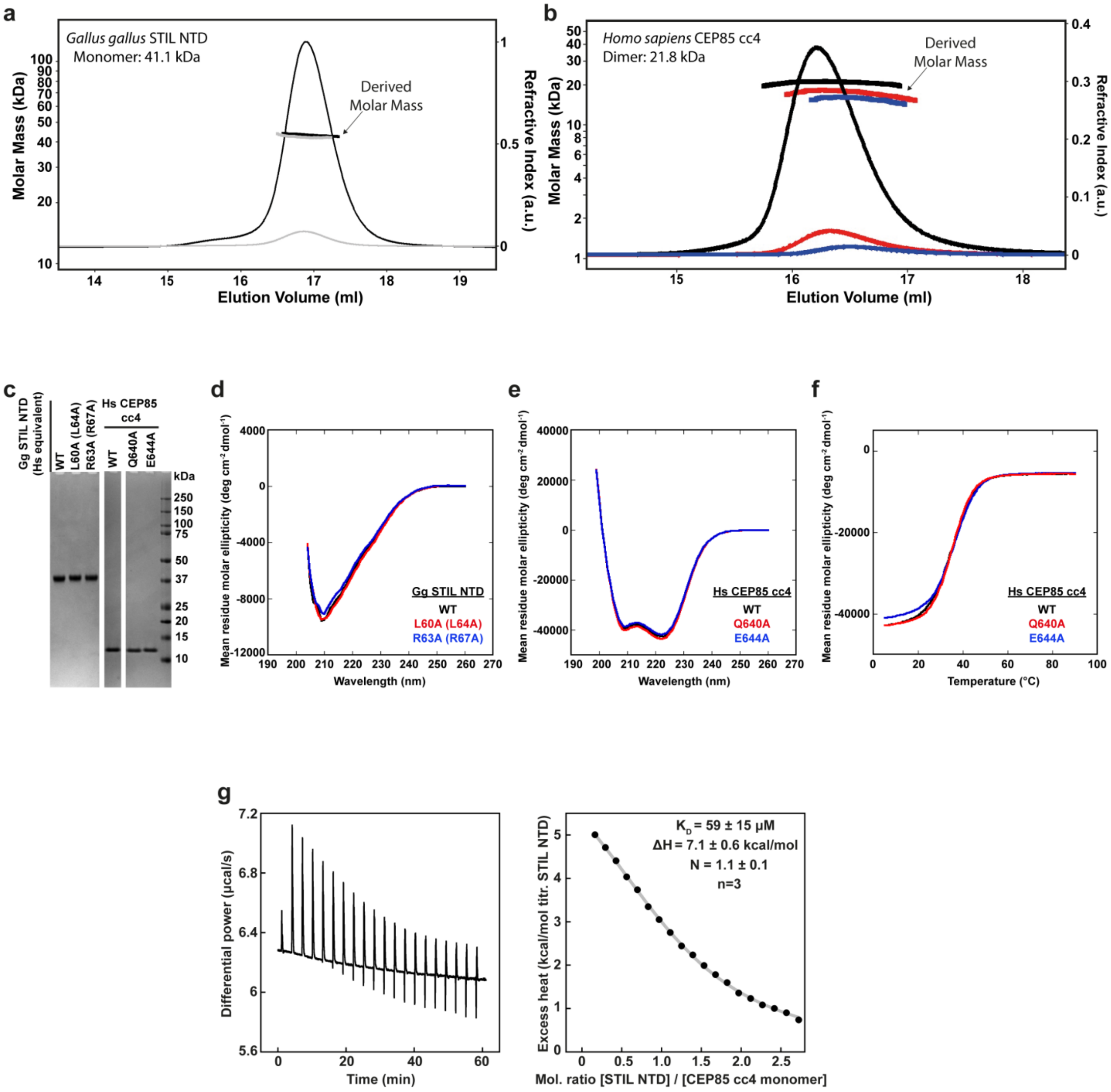

Supplementary Figure 5. Characterization of chicken STIL NTD, human CEP85 cc4 and their complex *in vitro*. (a, b). SEC-MALS chromatograms of recombinant chicken STIL NTD (a) and human CEP85 cc4 (b) run at varying concentrations. Shown are the respective refractive index signals together with the derived molar masses (indicated by thicker horizontal lines). The calculated, theoretical molecular weights are indicated. Chicken STIL NTD remained as a monomer over the concentration range examined while human CEP85 cc4 is clearly in a monomer-dimer equilibrium, only stably reaching the mass of the coiled-coil dimer at the highest loaded concentration examined. **(c).** Coomassie stained SDS-PAGE gels showing recombinant chicken STIL NTD (and mutants) and human CEP85 cc4 (and mutants) **(d, e).** CD analyses of recombinant chicken STIL NTD (d) and human CEP85 cc4 (e), WT and mutants. **(f).** CD-based thermal melting analysis of recombinant human CEP85 cc4, WT and mutants. **(g).** Recombinant chicken STIL NTD and human CEP85 cc4 directly interact with micromolar affinity and form a 2:2 complex. Typical ITC binding isotherm for chicken STIL NTD titrated into human CEP85 cc4 at 10 °C. The resulting K_D, ΔH and STIL NTD/CEP85 cc4 binding stoichiometry (N) as an average of three independent measurements are indicated (± standard deviation). At the used concentration and temperature of measurement, CEP85 cc4 appears fully folded (Supplementary Figure 5e, Supplementary Figure 5f), arguing that this interaction is driven by a large favourable entropy of binding while the endothermic enthalpy is unfavourable but smaller. In contrast, measurements at 25 °C (Figure 4D) apparently indicate that the binding is exothermic but these measurements might be distorted by the presence of low levels of unfolded CEP85 cc4 monomer in the ITC cell able to refold upon titration with the stabilising STIL NTD binding partner, thereby contributing a large exothermic heat effect. This effect could also affect the apparent stoichiometry of binding under these conditions that was lower than observed at 10 °C. Residue numbering is based on the used STIL homologues. Where applicable, the equivalent human STIL residue numbers are indicated in brackets.

Supplementary Figure 6

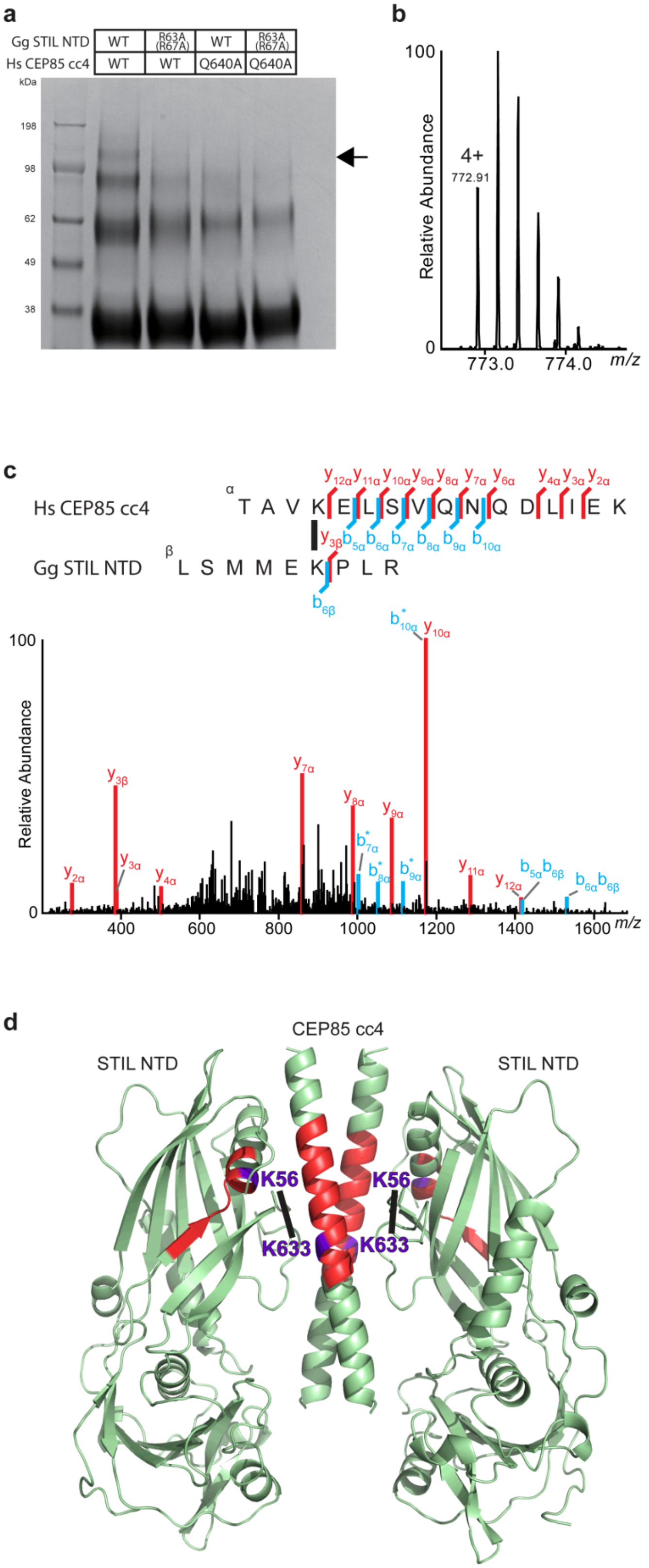

Supplementary Figure 6. Cross-linking mass-spectrometry analysis of chicken STIL NTD and human CEP85 cc4. (a). SDS-PAGE gel showing different combinations of wild-type and mutant recombinant chicken STIL NTD and human CEP85 cc4 after incubation with BS3 cross-linker. The arrow indicates the band corresponding to a STIL-NTD:CEP85 cc4 2:2 complex observed with the wild-type sample. **(b).** Mass spectrum of cross-linked peptides after in-gel digestion of the STIL-NTD:CEP85 cc4 complex shown as quaternary charge state. **(c).** MS/MS fragments of cross-linked peptides labeled on the sequence (top) and MS2 mass spectrum (bottom). The thick black line indicates the presence of a cross-link between K633 in human CEP85 and K56 in chicken STIL NTD. **(d).** Ribbon-presentation of a homology model of the human CEP85 cc4:chicken STIL NTD complex. The peptides identified in (c) are shown in red, the black line indicates the identified cross-link between STIL K56 and CEP85 K633 (colored in blue). The BS3 cross-linker has a spacer arm of 11.4 Å. Although side-chain densities (and therefore the corresponding Lysine ε-amino groups) were not resolved in the complex structure, the potential distance separating the cross-linked ε-amino groups of K633 and K56 appear compatible with this length (Cα distance between both residues is ~ 13.7 Å).

Supplementary Figure 7

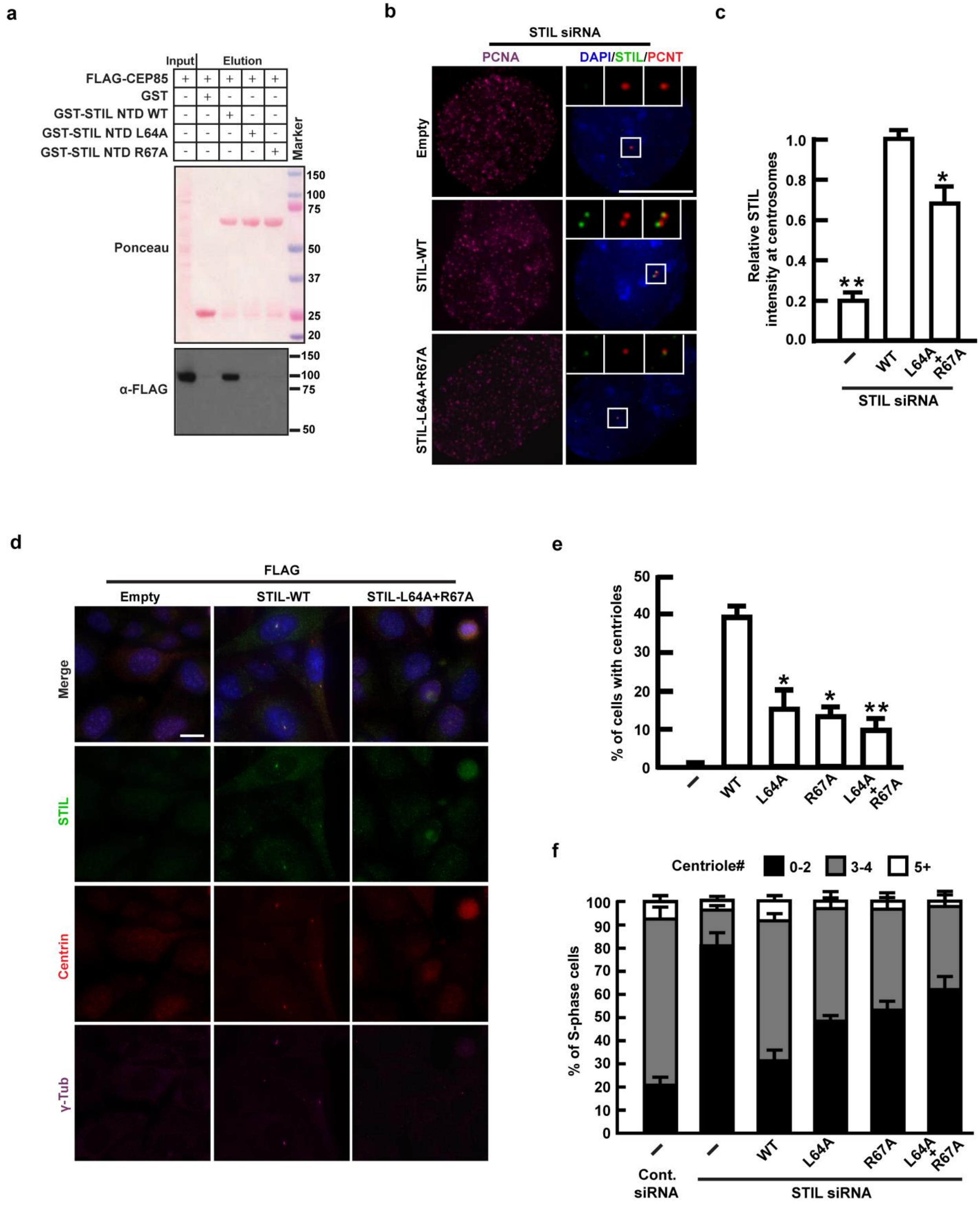

Supplementary Figure 7. The interaction between CEP85 and STIL is required for STIL localization and centriole assembly. (a). The interface residues described in Figure 5 are required for an efficient interaction of human STIL NTD and CEP85. Western blot showing a pull-down experiment with immobilized, recombinant GST or GST-human STIL NTD (WT and mutants), and lysates from tissue culture cells overexpressing 3xFLAG-tagged CEP85 as indicated. **(b, c).** The role of CEP85 binding in STIL centriolar localization. U-2 OS cells expressing Tet-inducible FLAG or the siRNA-resistant FLAG-STIL (WT and mutants) transgene were transfected with control or STIL siRNA for 72 h and tetracycline (2 μg/mL) was added for the final 48 h before fixation. The S-phase arrest assays were performed as described in Materials and Methods. Cells were labelled with DAPI and the indicated antibodies. Scale bar 10 μm, white boxes indicate the magnified region. **(c).** Quantification showing the relative levels of FLAG-STIL at centrosomes (n = 100/experiment, three independent experiments). **(d, e).** Effect of STIL mutations on *de novo* centriole formation. STIL CRISPR knockout U-2 OS cells were transfected with FLAG-STIL (WT and mutants) for 72 h. Selected images showing STIL, Centrin and γ-tubulin labelling. Scale bar 10 μm. **(e).** The graph shows the percentage of cells with restored centrioles (n = 100/experiment, three independent experiments). **(f).** Impact of STIL mutations on centriole duplication. U-2 OS cells conditionally expressing FLAG or siRNA-resistant FLAG-STIL (WT and mutants) were used to perform the S-phase arrest assays (See the Materials and Methods). Quantification showing the percentage of cells with the indicated centriole number. (n = 100/experiment, three independent experiments). Two-tailed t-test was performed for all p-values, all error bars represent S.D., and asterisks for p-values are **p<0.01 and *p<0.05.

Supplementary Figure 8

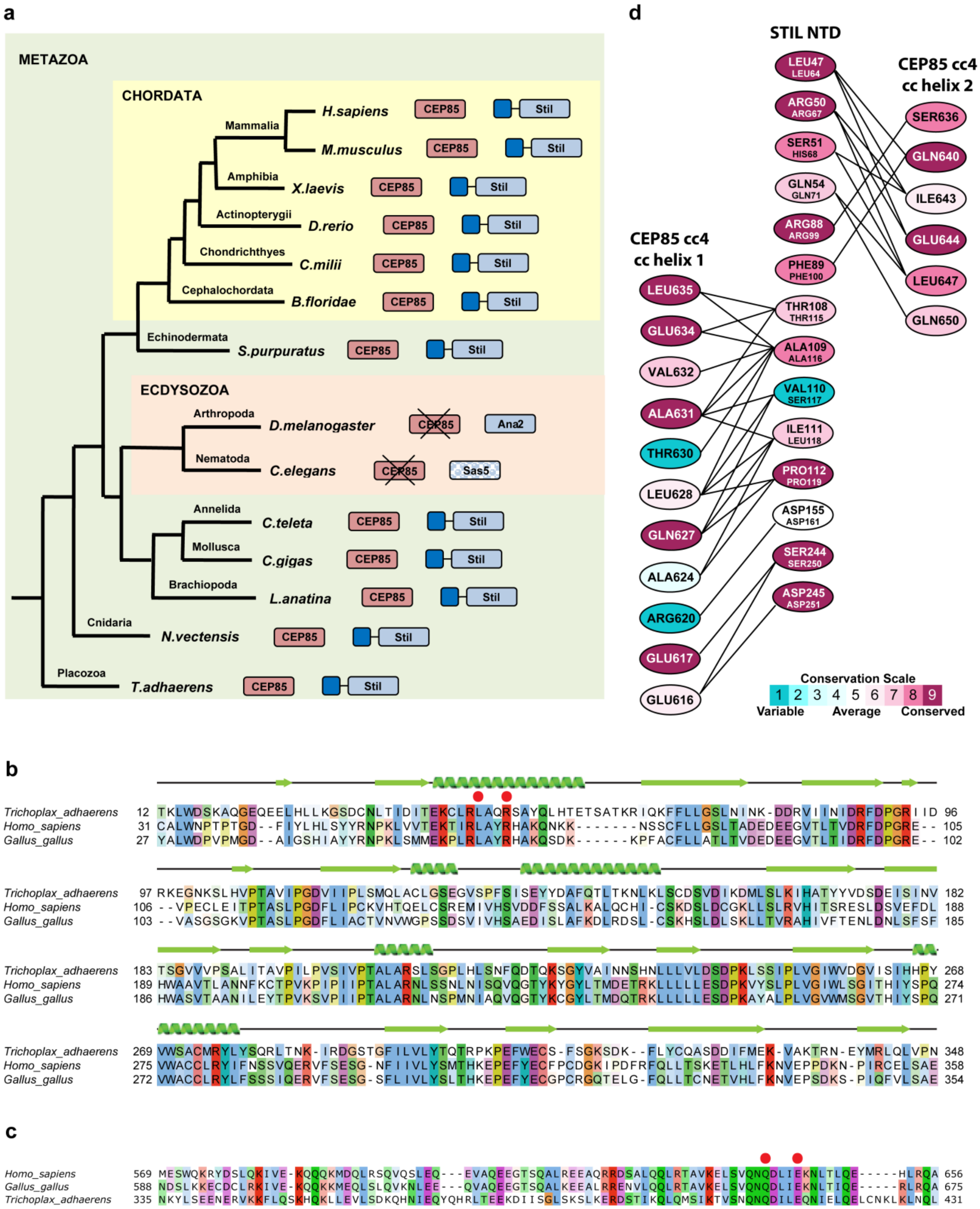

Supplementary Figure 8. Evolutionary conservation of CEP85 and STIL NTD. (a). Distribution of CEP85 and STIL across different representative metazoan organisms. CEP85 and STIL are shown as boxes, coloured in red and blue, respectively. The STIL NTD is shown as a dark blue box. Sas5 (STIL's functional homolog in *C. elegans*) is shown in a slightly different colour as its sequence does not have a detectable similarity to sequences of other STIL homologues. Insects such as flies, wasps, ants, butterflies, beetles and bees lack the STIL NTD. Their STIL domain organization and sequences are akin to *Drosophila* Ana2 (its STIL homologue). We were not able to identify CEP85 homologs in these organisms. **(b).** Multiple sequence alignment of the STIL NTD homologues from *Trichoplax adhaerens*, *Homo sapiens* and *Gallus gallus* that were used in this study. The alignment is coloured according to the CLUSTAL coloring scheme, residue color intensity is based on conservation. The secondary structure elements are shown above the alignment. The two STIL residues mutated in this study are indicated with a red dot. **(c).** Multiple sequence alignment of the CEP85 cc4 of CEP85 homologues from *Homo sapiens*, *Gallus gallus* and *Trichoplax adhaerens*. The alignment is coloured according to the CLUSTAL coloring scheme, residue color intensity is based on conservation. The two CEP85 residues mutated in this study are indicated with a red dot. **(d).** Conservation of the STIL NTD:CEP85 interaction interface. The resolution of our *Trichoplax adhaerens* STIL NTD: human CEP85 cc4 complex structure was insufficient to resolve side-chains. We defined the interaction interface as constituted by residues whose Cα distances between STIL NTD and CEP85 cc4 do not exceed 8 Å. The residues defining the interaction interface according to this criterion are shown in ellipses and are coloured by CONSURF conservation scores. Residue pairs located within an 8 Å distance are indicated with black lines. STIL residue numbers are according to *Trichoplax adhaerens* STIL NTD. The human equivalents of these residues are shown smaller below the *Trichoplax adhaerens* residues.

Supplementary Figure 9

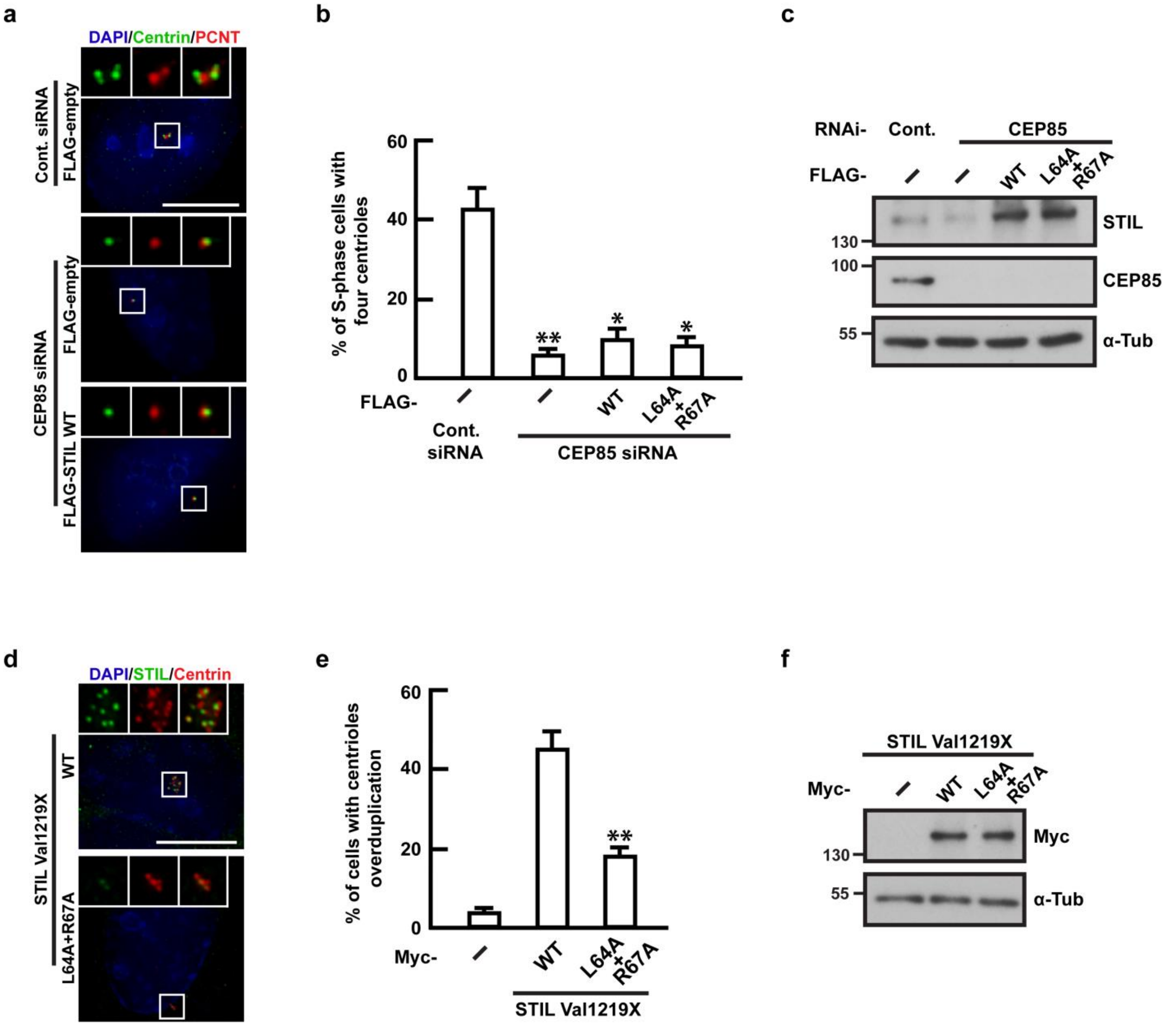

Supplementary Figure 9. STIL overexpression does not bypass the requirement for CEP85 in centriole duplication. (a). U-2 OS cells expressing Tet-inducible FLAG or the siRNA-resistant FLAG-STIL (WT and mutants) transgene were transfected with control or CEP85 siRNA for 72 h and tetracycline (2 μg/mL) was added for the final 48 h before fixation. The S-phase arrest assays were performed as described in Materials and Methods. Cells were labelled with DAPI and the indicated antibodies. Scale bar 10 μm, white boxes indicate the magnified region. **(b).** Quantification showing the percentage of cells with four centrioles (n = 100/experiment, three independent experiments). **(c).** Western blot showing the levels of CEP85 and STIL in control or CEP85 siRNA transfected cells. α-tubulin served as a loading control. **(d).** U-2 OS cells were transfected with Myc-STIL Val1219X (WT and mutants) for 48 h. Selected images showing Myc-STIL and Centrin labelling. Scale bar 10 μm, white boxes indicate the magnified region. **(e).** The graph shows the percentage of cells with over four centrioles (n = 100/experiment, three independent experiments). **(f).** Western blot showing the levels of Myc-STIL Val1219X (WT and mutants). α-tubulin served as a loading control. Two-tailed t-test was performed for all p-values, all error bars represent S.D., and asterisks for p-values are $^{**}p<0.01$ and $^{*}p<0.05$.

Supplementary Figure 10

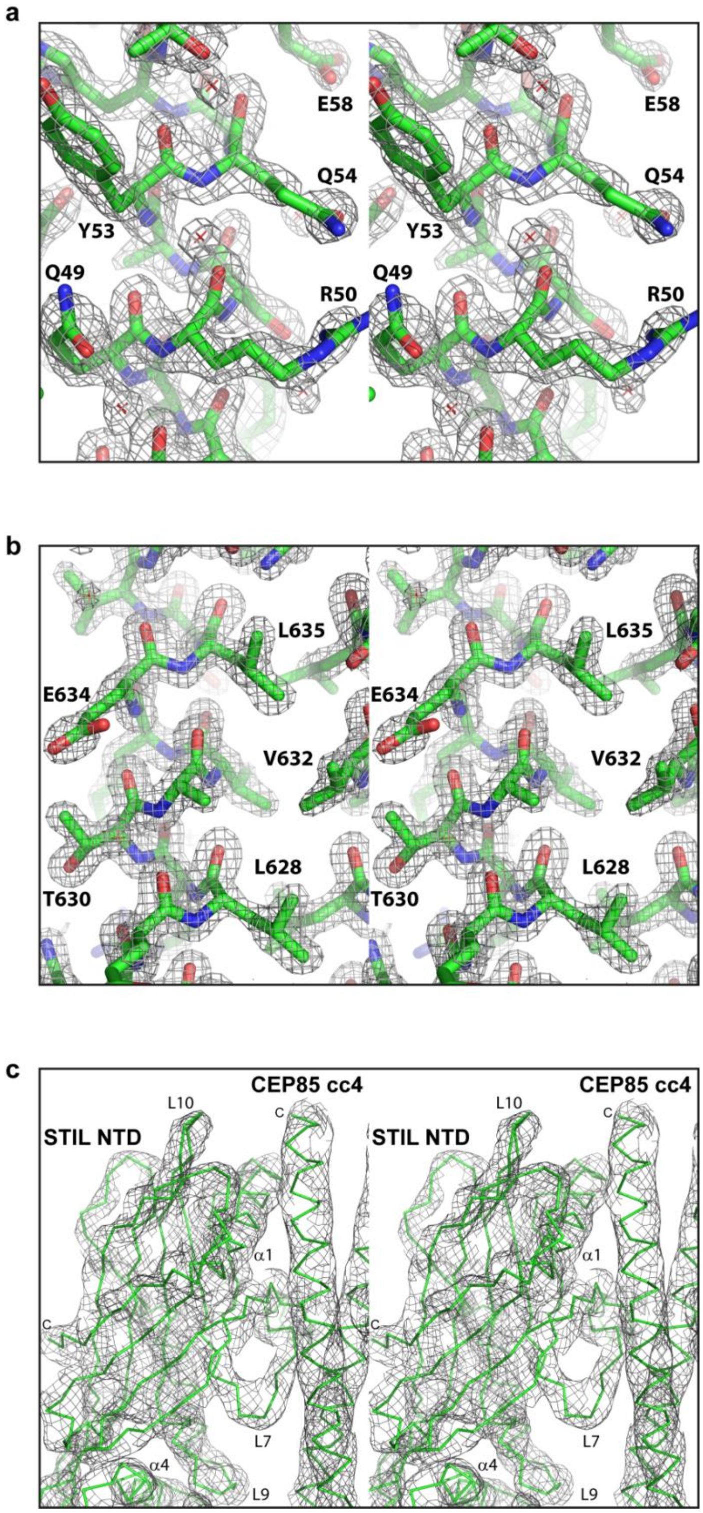

Supplementary Figure 10. **Stereo pictures of 2mFo-DFc electron density maps.**

2mFo-DFc electron density map (as iso-mesh representation at a contour level of σ=1.5) of the reported X-ray crystallographic structures of (**a**) *Trichoplax adhaerens* STIL NTD, (**b**) human CEP85 cc4 and **c**) the *Trichoplax adhaerens* STIL NTD : human CEP85 cc4 complex. Models are shown in sticks (a,b) or as ribbon presentation (c). Selected residues (a, b) or secondary structure elements (c) are labelled to aid orientation.

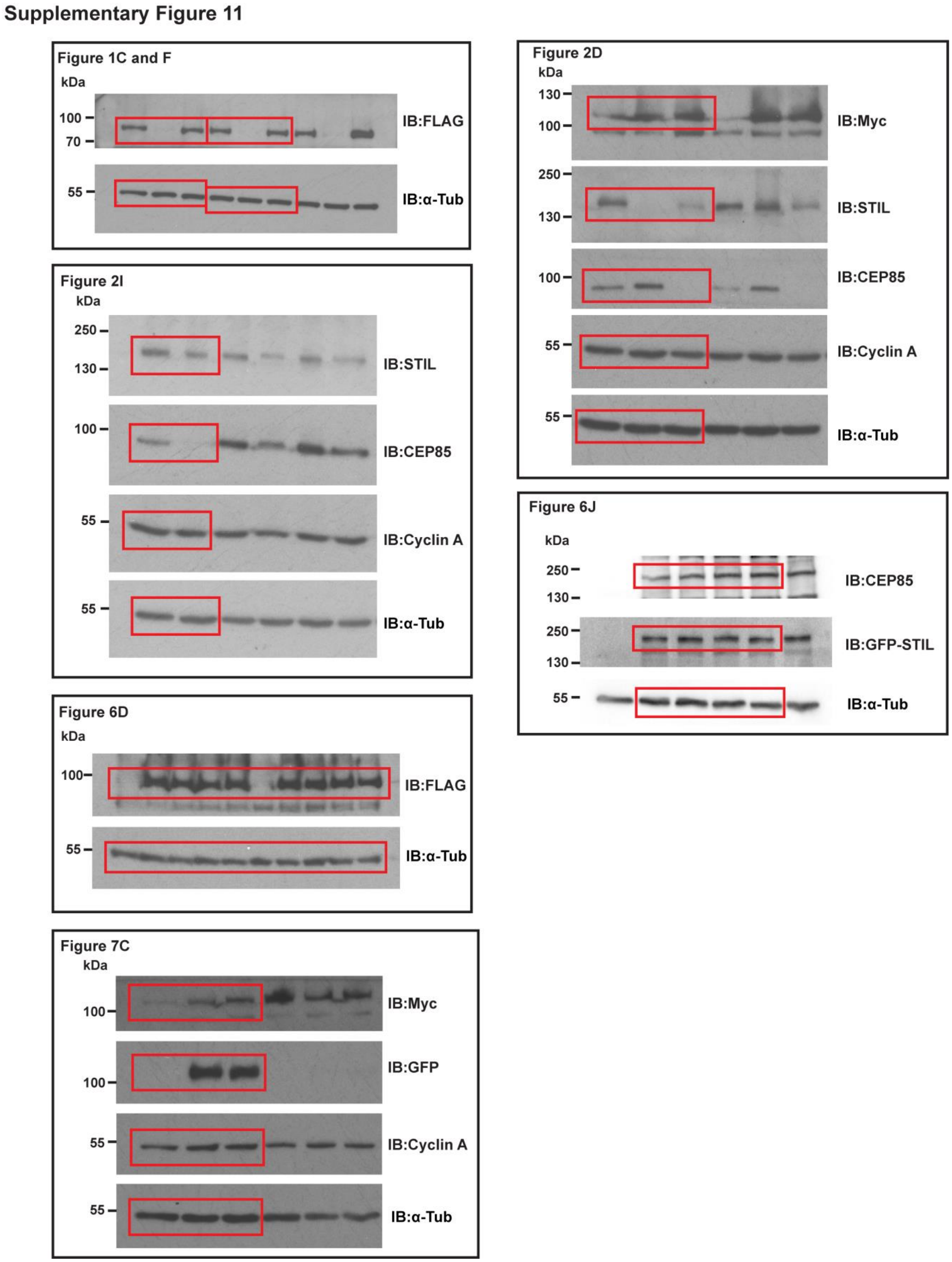

Supplementary Figure 11. Uncropped scans of the western blots shown in Figure 1C and F, Figure 2D and I, Figure 6D and J and Figure 7C.

Supplementary Figure 12

Figure 4B

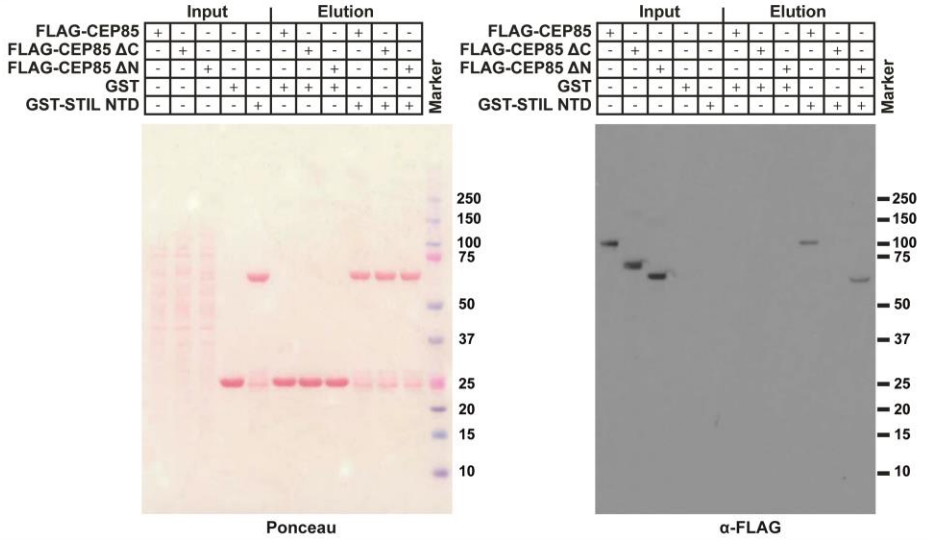

Figure 6A

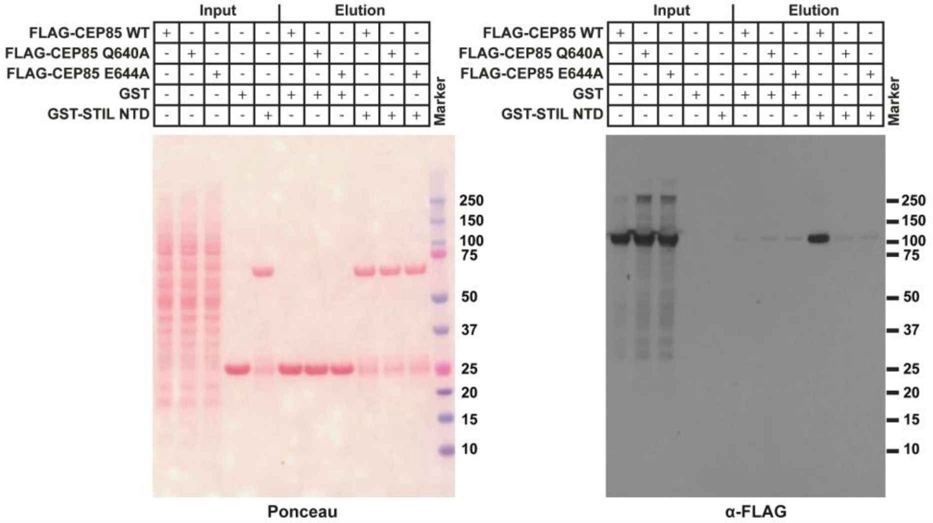

Supplementary Figure 7a

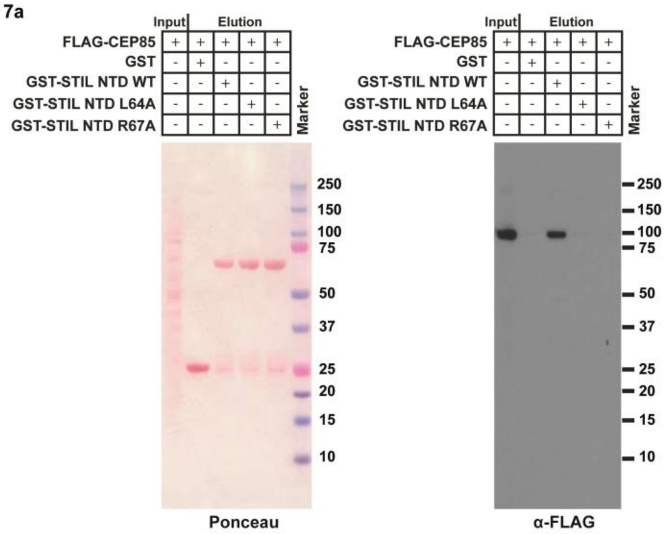

Supplementary Figure 12. Uncropped scans of the western blots shown in Figure 4B, Figure 6A and Supplementary Figure 7a.

Supplementary Figure 13

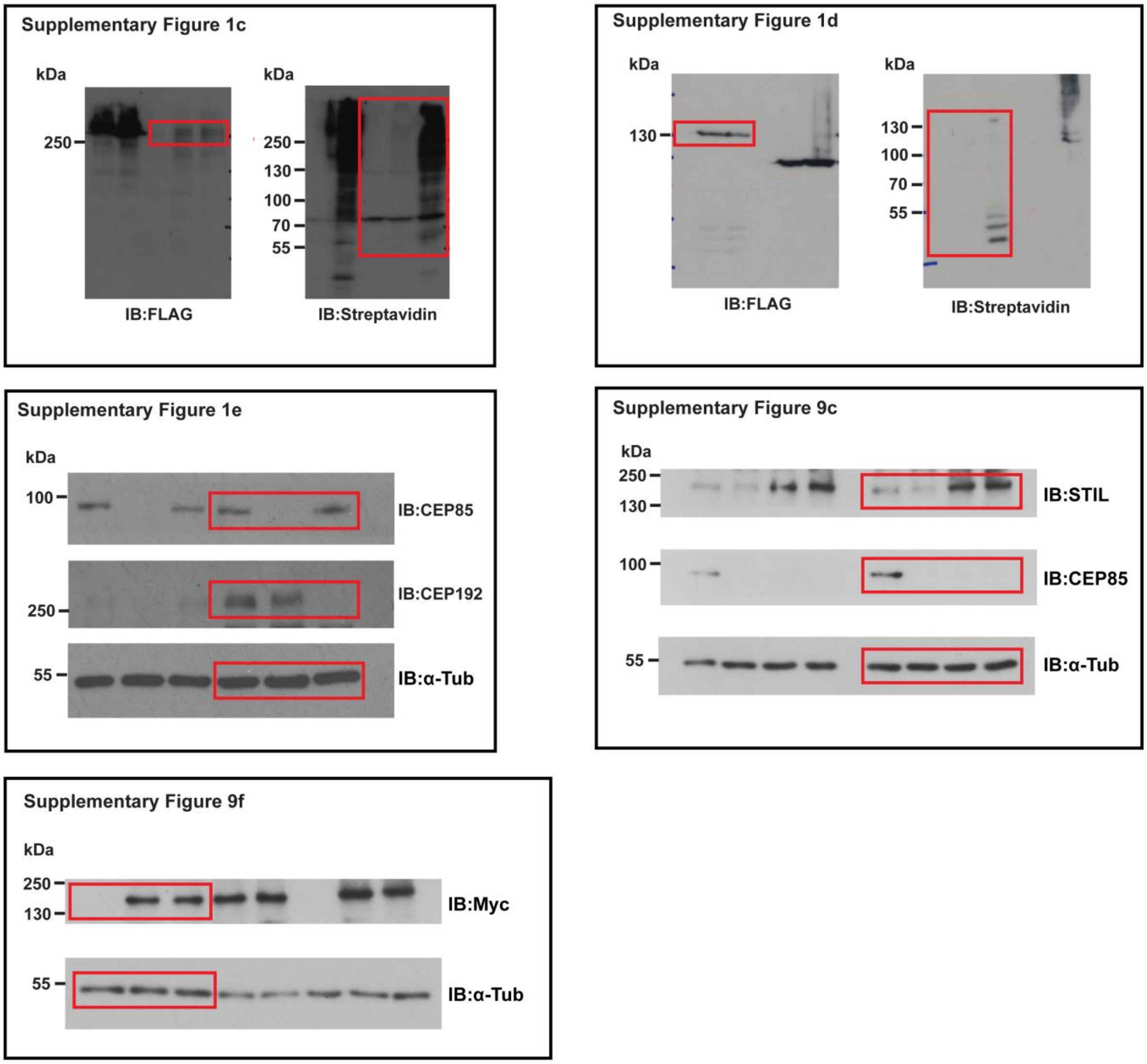

Supplementary Figure 13. Uncropped scans of the western blots shown in Supplementary Figure 1c, d and e and Supplementary Figure 9c and f.

Supplementary Table 1. Data collection statistics.

	SeMet *T. adhaerens* STIL[1-348]	
**Data collection**		
Space Group	$P2_1$	
	Peak	Remote
Cell dimensions a,b,c (Å) α, β, γ (°)	65.9, 75.1, 68.6<br>90.0, 97.0, 90.0	66.0, 75.2, 68.5<br>90.0, 97.2, 90.0
Wavelength (Å)	0.97916	0.87290
Resolution (Å)	75.18 - 2.09<br>(2.14 - 2.09)	37.59 - 2.09<br>(2.14 - 2.09)
R_{merge}	0.167 (0.845)	0.205 (1.004)
R_{pim}	0.103 (0.520)	0.088 (0.429)
$I/\sigma I$	7.8 (2.6)	8.0 (2.2)
Completeness (%)	91.2 (90.9)	99.9 (99.3)
Redundancy	3.4 (3.4)	6.3 (6.2)

Footnote: Datasets for each wavelength derive from single crystals

Supplementary Table 2. Data collection statistics.

	Complex (SeMet) *T. adhaerens* STIL[1-348] - *H.sapiens* CEP85[570-656]		
**Data collection**			
Space Group	$I4_132$		
	Peak	Inflection	Remote
Cell dimensions a,b,c (Å) α, β, γ (°)	269.2, 269.2, 269.2<br>90.0, 90.0, 90.0	269.9, 269.9, 269.9<br>90.0, 90.0, 90.0	268.3, 268.3, 268.3<br>90.0, 90.0, 90.0
Wavelength (Å)	0.97924	0.97931	0.93927
Resolution (Å)	49.13 - 5.00<br>(5.59 - 5.00)	49.13 - 5.00<br>(5.59 - 5.00)	49.13 - 5.00<br>(5.59 - 5.00)
R_{merge}	0.696 (18.568)	0.537 (10.903)	0.245 (4.926)
R_{pim}	0.109 (2.938)	0.082 (1.672)	0.038 (0.751)
$I/\sigma I$	7.6 (0.6)	7.5 (0.6)	9.4 (1.2)
Completeness (%)	99.9 (100.0)	99.9 (100.0)	99.9 (100.0)
Redundancy	41.9 (40.4)	42.7 (42.7)	43.0 (43.3)

Footnote: Datasets for each wavelength derive from single crystals

Supplementary Table 3. Data collection statistics.

	Complex (SeMet) *T. adhaerens* $STIL^{1\text{-}348}$ – *H.sapiens* $CEP85^{570\text{-}656}$ T630M / H652M	
**Data collection**		
Space Group	$I4_132$	
	$Cep85^{570\text{-}656}$ T630M (Peak)	$Cep85^{570\text{-}656}$ H652M (Peak)
Cell dimensions a,b,c (Å) α, β, γ (°)	268.6, 268.6, 268.6<br>90.0, 90.0, 90.0	269.4, 269.4, 269.4<br>90.0, 90.0, 90.0
Wavelength (Å)	0.97911 (Peak)	0. 97911 (Peak)
Resolution (Å)	47.47 - 5.00 (5.59 - 5.00)	47.62 - 5.00 (5.59 - 5.00)
R_{merge}	0.457 (5.283)	0.447 (6.548)
R_{pim}	0.070 (0.830)	0.069 (1.017)
$I/\sigma I$	10.6 (1.6)	10.9 (1.2)
Completeness (%)	99.9 (100.0)	99.9 (100.0)
Redundancy	42.7 (42.0)	42.5 (41.8)

Footnote: Datasets derive from single crystals

Supplementary Table 1-3.

Data collection statistics of the two-wavelength MAD datasets of selenomethionine *T. adhaerens* $STIL^{1\text{-}348}$ (Supplementary Table 1), of the three-wavelength MAD datasets of the selenomethionine *T. adhaerens* $STIL^{1\text{-}348}$ - *H.sapiens* $CEP85^{570\text{-}656}$ complex (Supplementary Table 2) and of the selenium peak wavelength datasets of the selenomethionine *T. adhaerens* $STIL^{1\text{-}348}$ - *H.sapiens* $CEP85^{570\text{-}656}$ T630M / H652M complexes (Supplementary Table 3). Values in parenthesis are for highest resolution shell.

Supplementary Methods

Recombinant protein purification

DNA encoding *T. adhaerens* STIL$^{1-348}$ or *G. gallus* STIL$^{14-369}$ (WT and mutants) were cloned into a modified pRSETa vector (Invitrogen) containing two His-tagged lipoyl domains [3]. SeMet *T. adhaerens* STIL$^{1-348}$ and *G. gallus* STIL$^{14-369}$ were subsequently expressed in *E.coli* C41(DE3) either in supplemented M9 medium [4] (SeMet *T. adhaerens* STIL$^{1-348}$) or in 2xTY medium (*G. gallus* STIL$^{14-369}$) and purified by standard methods using NiNTA (Qiagen) beads. After elution, the eluates were dialysed against 50 mM Tris-HCl, pH 8.0, 300 mM NaCl, 5 mM imidazole, pH 7.5, 10 mM β-mercapto-ethanol in the presence of TEV protease (kind gift of Mark Allen, MRC-LMB, Cambridge, UK) to cut off the His-tagged lipoyl tags and rebound to NiNTA Agarose (Qiagen) to remove the tags. The flow-through was further purified by size exclusion chromatography in 10 mM Tris-HCl, pH 8.0, 50 mM NaCl, 1 mM DTT (SeMet *T. adhaerens* STIL$^{1-348}$) or by ion-exchange chromatography (HiTrap Q-FF, GE Healthcare) using linear salt gradients from 10 mM Tris-HCl, pH 8.0, 2 mM DTT to 10 mM Tris-HCl, pH 8.0, 2 mM DTT, 1 M NaCl (*G. gallus* STIL$^{14-369}$).

DNA encoding *H.sapiens* CEP85$^{570-656}$ or CEP85$^{570-662}$ (WT and mutants) were cloned into a modified pOPTH vector [5] giving rise to a N-terminally His and MBP-tagged construct. Constructs were expressed in *E.coli* C41 (DE3) and purified by NiNTA (Qiagen) chromatography using standard methods. The eluates were dialysed against 50 mM Tris-HCl, pH 8.0, 300 mM NaCl, 5 mM imidazole, pH 7.5 in the presence of TEV protease (kind gift of Mark Allen, MRC-LMB, Cambridge, UK) to cut off the His-MBP tag, rebound to NiNTA Agarose (Qiagen) to remove the tag and the flow-throughs further purified by size exclusion

chromatography in 10 mM Tris-HCl, pH 7.4 (CEP85$^{570-656}$) or pH 8.0 (CEP85$^{570-662}$), 50 mM NaCl followed by ion-exchange chromatography (HiTrap Q-FF, GE Healthcare) using linear salt gradients from 10 mM Tris-HCl, pH 8.0 to 10 mM Tris-HCl, pH 8.0, 1 M NaCl (CEP85$^{570-662}$).

The SeMet *T. adhaerens* STIL$^{1-348}$ - *H.sapiens* CEP85$^{570-656}$ complex was purified by mixing the SeMet *T. adhaerens* STIL$^{1-348}$ and the *H.sapiens* CEP85$^{570-656}$ expressing cell cultures, followed by cell lysis, NiNTA purification, TEV cleavage and removal of the cut-off tags by rebinding to NiNTA resin as described above. The flow-through was dialysed against 10 mM Tris-HCl, pH 8.0, 2 mM DTT, NaCl added to 75 mM and the complex purified further by ion-exchange chromatography from 10 mM Tris-HCl, pH 8.0, 2 or 4 mM DTT to 10 mM Tris-HCl, pH 8.0, 1 M NaCl, 2 or 4 mM DTT (linear gradient) on a Mono-Q FPLC column (Pharmacia).

DNA encoding human STIL$^{1-390}$ (WT and mutants) (including an N-terminal linker (SSRSNQTSLYKKAGSAAAPFT), were cloned into the BamH1 and EcoR1 sites of pGEX-6P1 (GE Healthcare). Constructs (and pGEX-6P1 alone) were expressed in *E.coli* Rosetta at 18°C and purified by Glutathione Sepharose 4B resin (GE Healthcare) and ion exchange chromatography (HiTrap Q HP column, GE Healthcare) using a linear salt gradient from 10 mM Tris-HCl, pH 8.5, 2 mM DTT to 10 mM Tris-HCl, pH 8.5, 1 M NaCl, 2 mM DTT. All proteins were concentrated, flash-frozen in liquid nitrogen and stored at -80°C.

Data collection and processing

The SeMet *T. adhaerens* STIL$^{1-348}$ datasets were collected at 100K to a resolution of 2.1 Å at beamline ESRF ID23-2 at a wavelength of 0.8729 Å (remote) or at beamline ESRF ID29 at a wavelength of 0.97916 Å (peak). The datasets of the SeMet *T. adhaerens* STIL$^{1-348}$-*H.sapiens*

CEP85$^{570-656}$ complex were collected at 100K to a resolution of 4.6 or 5 Å at beamline ESRF ID29 at a wavelength of 0.97924 Å (peak), 0.97931 Å (inflection) and 0.93927 Å (remote). The datasets of the SeMet *T. adhaerens* STIL$^{1-348}$-*H.sapiens* CEP85$^{570-656}$ T630M or H652M complex were collected to a resolution of 5 Å at 100K at beamline ESRF ID29 at a wavelength of 0.97911 Å. The *H.sapiens* CEP85$^{570-656}$ dataset was collected to a resolution of 1.7 Å using an in-house source (Rigaku FR-E+ SuperBright) at a wavelength of 1.54179 Å and a temperature of 100K.

Datasets were integrated using MOSFLM [6] (SeMet *T. adhaerens* STIL$^{1-348}$, *H.sapiens* CEP85$^{570-656}$, SeMet *T. adhaerens* STIL$^{1-348}$-*H.sapiens* CEP85$^{570-656}$ complex (remote dataset used for refinement)) or XDS [7] (SeMet *T. adhaerens* STIL$^{1-348}$-*H.sapiens* CEP85$^{570-656}$ complex MAD dataset, SeMet *T. adhaerens* STIL$^{1-348}$-*H.sapiens* CEP85$^{570-656}$ T630M and H652M). Datasets were scaled using SCALA [8] (*H.sapiens* CEP85$^{570-656}$), or AIMLESS [9] (SeMet *T. adhaerens* STIL$^{1-348}$ datasets, SeMet *T. adhaerens* STIL$^{1-348}$-*H.sapiens* CEP85$^{570-656}$ complex (and mutants)).

The SeMet *T. adhaerens* STIL$^{1-348}$ structure was solved from a 2-wavelength MAD dataset using the CRANK pipeline [10], resulting in an easily interpretable experimental electron density map and the successful identification of all ten expected selenium sites (10 seleno-methionines are visible in the structure). The structure of *H.sapiens* CEP85$^{570-656}$ was solved by molecular replacement in PHASER [11] using a parallel coiled-coil dimer model (as Poly-Ala) derived from PDB 2Q6Q. Initial models of SeMet *T. adhaerens* STIL$^{1-348}$ and *H.sapiens* CEP85$^{570-656}$ were built using BUCCANEER [12] and manual building in COOT [13] and both structures were refined in PHENIX.REFINE [14] with manual building performed in COOT [13].

The SeMet *T. adhaerens* STIL$^{1-348}$-*H.sapiens* CEP85$^{570-656}$ complex structure was solved from a three-wavelength MAD dataset using the SHELX CDE pipeline [15], resulting in an

experimental electron density map that could readily be interpreted (Supplementary Figure S4a). Into this experimental electron density map, the structure of *T. adhaerens* STIL$^{1-348}$ was manually placed using the positions of the five detected Selenium sites within the density corresponding to *T. adhaerens* STIL$^{1-348}$ as marker sites (five seleno-methionines are visible in the structure). Although clear electron density corresponding to the *H.sapiens* CEP85$^{570-656}$ coiled-coil structure was present (Supplementary Figure 4a), due to a lack of methionines in this part of the structure, the register and orientation of the coiled-coil was unclear. To obtain these, the phases from the experimental electron density map of the SeMet *T. adhaerens* STIL$^{1-348}$-*H.sapiens* CEP85$^{570-656}$ complex were combined with the amplitudes of collected peak wavelength datasets from crystals of the corresponding SeMet *T. adhaerens* STIL$^{1-348}$-*H.sapiens* CEP85$^{570-656}$ T630M or H652M mutant complexes. From these datasets, phased anomalous difference maps were calculated using FFT [16], resulting in clear extra selenium densities corresponding to the T630M and the H652M positions (Supplementary Figure 4b) that were subsequently used to place the *H.sapiens* CEP85$^{570-656}$ structure. Refinement was done in REFMAC [17] and PHENIX.REFINE [14] using secondary structure and Ramachandran restraints together with reference model restraints from the apo-SeMet *T. adhaerens* STIL$^{1-348}$ structure and the placed *H.sapiens* CEP85$^{570-656}$ structure. Due to the obtained resolution (4.6 Å), no side-chain densities were visible in the complex structure and therefore a final refinement run was done with a Poly-Ala model of the complex. Manual building was done in COOT [13].

Electron density maps of the reported X-ray crystallographic structures are shown in Supplementary Figure 10.

GST-STIL NTD - 3xFLAG-CEP85 pull-down assay

This assay was adapted from [18]. ~30 μg of purified GST or GST-*H.sapiens* STIL$^{1-390}$ were bound to Glutathione Sepharose 4B (GE Healthcare) beads in Lysis buffer (50 mM Bis-Tris-Propane, pH 7.2, 100 mM NaCl, 1 mM DTT, 0.1% (v/v) Nonidet-P40, supplemented with Complete Protease Inhibitor (EDTA free, Roche), washed and subsequently incubated (4 °C, 1 h) with centrifugationally cleared cell lysates (obtained by sonication) from Hek293 Trex Flpin cells (kind gift of Manu Hegde, MRC-LMB, Cambridge, UK) transfected for ~2 days by Fugene 6 or Fugene HD (Promega) with 3xFLAG human CEP85 constructs (cloned into a pcDNA3.1 derivative, kind gift of Manu Hegde, MRC-LMB, Cambridge, UK). After washing with Lysis buffer, beads were eluted (Lysis buffer, 100 mM Glutathione, pH 7.5) and eluates separated by SDS-PAGE before Western Blotting with anti-FLAG M2 mouse monoclonal antibody (Sigma). Uncropped scans of the corresponding Western blots are shown in Supplementary Figure 12.

Yeast Two Hybrid

Constructs were cloned into vector pENTR/D-TOPO and transferred into vectors pDEST32 (Bait, human STIL NTD) or pDEST22 (Prey, human CEP85 constructs) using site-specific recombination (Gateway LR Clonase II, Thermo Fisher Scientific). Bait and Prey plasmid combinations were co-transformed into yeast strain MaV203 (Thermo Fisher Scientific) and plated onto SC-Leu-Trp plates. Colonies were inoculated into SC-Leu-Trp medium, grown and spotted onto SC-Leu -Trp and SC-Ura plates. Plates were incubated for three days at 30°C.

Native mass spectrometry

Purified *G. gallus* STIL$^{14-369}$ and *H.sapiens* CEP85$^{570-662}$ protein solutions (in 100 mM ammonium acetate pH 7.5, 1 mM DTT) were diluted to 40 μM protein concentration separately

or mixed at equivalent molar ratio to a final protein concentration of 40 µM using 100 mM ammonium acetate solution. The diluted protein samples were further buffer exchanged into 100 mM ammonium acetate, pH 7.3, using a micro biospin column (Micro Bio-Spin 6, Bio-Rad) before native MS analysis. *G. gallus* STIL$^{14\text{-}369}$- *H.sapiens* CEP85$^{570\text{-}662}$ complex and *G. gallus* STIL$^{14\text{-}369}$ alone experiments were conducted using a Q Exactive instrument (Thermo Fisher, Germany) with modifications for high-mass transmission optimisation. The instrument was calibrated using cesium iodide solution. 2 µl of buffer exchanged protein solution was electrosprayed from gold-plated borosilicate capillaries prepared in house. The following instrument parameters were used: 1.2 kV capillary voltage, 100 V S-lens and 30 °C capillary temperature. Pressure in the HCD cell was raised to $1.2e^{-9}$ mbar for efficient transmission of protein ions. *H.sapiens* CEP85$^{570\text{-}662}$ sample was analysed using a Synapt G1 mass spectrometer (Waters) modified for the transmission of intact protein ions under the following conditions: capillary, cone and collision cell at 1.2 kV, 20 V and 20 V, respectively. The mass spectra were analysed and mass assigned using the UniDec program [19].

Cross-linking and LC-MS

The protein cross-linking method was adapted from[20]. Protein samples were buffer-exchanged to phosphate buffered saline (PBS) (pH 7.4) using micro biospin columns (Micro Bio-Spin 6, Bio-Rad) to remove free amines. Two runs were performed for a complete buffer exchange. Human CEP85$^{570\text{-}662}$ cc4 (WT or Q640A mutant) and chicken STIL$^{14\text{-}369}$ NTD (WT or R63A) were then mixed to a final 40 µM protein concentration. To initiate cross-linking, BS3-d0 cross-linker (Thermo Fisher Scientific) was added to the protein samples at a final concentration of 0.8 mM and incubated at 4 °C for 2h. The excessive BS3-d0 in the samples

was quenched by adding 1 M Tris·HCl (pH 8.0) to a final concentration of 50 mM. The cross-linked protein samples were separated by SDS-PAGE. The CEP85-STIL tetramer gel band was cut and digested with trypsin[21]. The tryptic peptides were extracted by typical extractions methods[22] and analyzed on EASY-nLC 1000 (Thermo Fisher Scientific) coupled to LTQ-Orbitrap XL spectrometer (Thermo Fisher Scientific) via a dynamic nanospray source. The fragmentation pattern of the peptide of interest was manually analysed.

Analytical Ultracentrifugation (AUC)

Samples of *G. gallus* STIL$^{14\text{-}369}$ and *H.sapiens* CEP85$^{570\text{-}662}$ in 50 mM Tris-HCl, pH 7.4, 100 mM NaCl, 1 mM DTT were subjected to equilibrium sedimentation in an An50Ti rotor using an Optima XL-I analytical ultracentrifuge (Beckmann). The partial-specific volumes (v-bar), solvent density and viscosity were calculated using Sednterp (Dr. Thomas Laue, University of New Hampshire). Sample volumes of 110 µL with total protein concentrations of: 230, 76 and 26 µM for *H.sapiens* CEP85$^{570\text{-}662}$ alone; 360, 120, and 40 µM for *G. gallus* STIL$^{14\text{-}369}$ both alone and in combination with an equal concentration of *H.sapiens* CEP85$^{570\text{-}662}$ were loaded in 12 mm 6-sector cells and centrifuged at 6,500, 17,400, and 30,000 rpm until equilibrium was reached at 10 ˚C. At each speed, comparison of several scans was used to judge whether or not equilibrium had been reached. Data were processed and analysed using SEDPHAT [23].

Isothermal Titration Calorimetry (ITC)

ITC measurements were performed using an Auto iTC200 instrument (Malvern Instruments, Malvern, UK) at 10 and 25°C using 50 mM Tris-Cl, pH 7.4, 100 mM NaCl, 1 mM DTT buffer. Samples of *H.sapiens* CEP85$^{570\text{-}662}$ at ~ 100 µM in the calorimeter cell were titrated

with *G. gallus* $STIL^{14\text{-}369}$ at ~ 1 mM from the syringe using 19 injections of 2 µL preceded by a small 0.5 µL injection to eliminate pre-mixed material which can accumulate in the end of the syringe needle during the pre-measurement equilibration phase. The resulting excess heats associated with each 2 µL injection were integrated and normalized using the measured concentrations of protein from UV absorbance. Measurements were corrected for the background heat of dilution of *G. gallus* $STIL^{14\text{-}369}$ using controls injecting equivalent concentration of protein into buffer alone and data were finally fit to a simple single site binding model using the PEAQ Analysis software (Malvern Instruments, Malvern, UK).

The stoichiometry of binding at 25°C was somewhat ambiguous (N=0.34 ± 0.06, N=7) but we were unable to perform the potentially informative reverse titration, with *H.sapiens* $CEP85^{570\text{-}662}$ in the syringe, because of its limited stability at 25°C (Supplementary Figure 5b and Supplementary Figure 5f). Indeed, control ITC titrations of *H.sapiens* $CEP85^{570\text{-}662}$ from the syringe into buffer produced a typical saturating endothermic thermogram that is commonly seen for an equilibrium dissociating system when the K_D is such that the concentration in the syringe produces stable oligomer while that in the cell after injection leads to some dissociation; in this case the strands of the CEP85 coiled-coil. We therefore also performed ITC experiments at 10 °C where the *H.sapiens* $CEP85^{570\text{-}662}$ coiled coil dimer is more stable and confirmed the expected stoichiometry of binding at 2 : 2, *G. gallus* $STIL^{14\text{-}369}$: *H.sapiens* $CEP85^{570\text{-}662}$ (i.e. two bound *G. gallus* $STIL^{14\text{-}369}$ per *H.sapiens* $CEP85^{570\text{-}662}$ coiled coil dimer) (Supplementary Figure 5g).

Circular Dichroism (CD)

Far-UV CD spectra were measured in PBS buffer in a 1 mm pathlength cuvette using a Jasco J815 spectropolarimeter (Jasco, Great Dunmow, UK). Samples were diluted to a final

concentration of 0.55 mg/mL (*H.sapiens* CEP85$^{570\text{-}662}$ and mutants) or 0.62 mg/mL (*G. gallus* STIL$^{14\text{-}369}$ and mutants) and spectra measured as the average of 8 or 16 rescan acquisitions respectively. Spectra of *H.sapiens* CEP85$^{570\text{-}662}$ and mutants were recorded at 5 °C (Supplementary Figure 5e) before monitoring thermal denaturation of the coiled coil structure at a single wavelength of 222nm using a heating rate of 60 °C / hr (Supplementary Figure 5f). All spectra were corrected for the measured PBS buffer baseline.

Size exclusion chromatography with multi-angle light scattering (SEC MALS)

The mass of *H.sapiens* CEP85$^{570\text{-}662}$ and *G. gallus* STIL$^{14\text{-}369}$ in solution was determined using SEC MALS. Samples for analysis in a standard SEC MALS format were first resolved on a GE Healthcare Superdex S200 10/300 column running at 0.5 mL/min before detection using a Wyatt Heleos II 18 angle light scattering light scattering instrument followed by a Wyatt Optilab rEX refractive index instrument. Protein concentration was determined from the excess differential refractive index using ΔRI of 0.186 for 1 g/ml and used with the observed scattered intensity to calculate the absolute molecular mass from the intercept of a Debye plot using Zimm's model as implemented in ASTRA software from Wyatt. The Heleos instrument included a dynamic light scattering detector at 99° angle from which a translational diffusion coefficient could be evaluated by autocorrelation analysis of intensity fluctuations assuming a single diffusing species. This was used to calculate the hydrodynamic radius using the Stokes-Einstein equation and the measured solvent viscosity of 9.3 e-3 Poise.

Buffer for *H.sapiens* CEP85$^{570\text{-}662}$, loaded between 5 and 0.2 mg/mL, was 50 mM Tris-Cl, pH 7.4, 100 mM NaCl while for *G. gallus* STIL$^{14\text{-}369}$, loaded between 17 and 1 mg/mL, it

was 50 mM Bis-Tris-Propane, pH 7.2, 100 mM NaCl. Typically, the maximum concentration obtained during the SEC MALS measurements was ~ 1/10 of the loaded concentration due to dilution during SEC.

NMR

All NMR data were collected at 298K on a Bruker Avance II+ 700 MHz spectrometer, equipped with cryogenic triple-resonance TCI probe. 2D ^{15}N,^{1}H BEST-TROSY datasets for 75 μM ^{15}N labelled wild-type *G. gallus* $STIL^{14-369}$ and R63A mutant alone and in the presence of unlabelled wild-type *H.sapiens* $CEP85^{570-662}$ were acquired in 50 mM $NaPO_4$ buffer, 100 mM NaCl and 1 mM DTT, pH 7.5. Data were processed using Topspin 3.0 (Bruker) and analyzed using SPARKY (T. D. Goddard and D. G. Kneller - University of California, San Francisco).

Bioinformatics

NCBI-NR was searched using PSI-BLAST [24] to identify sequence homologs of CEP85 and STIL. Selected sequences of CEP85 and STIL homologs were aligned; the alignments were corrected manually and then used as input for calculation of conservation scores using CONSURF [25]. Multiple sequence alignments were produced with MAFFT [26] and visualized using JALVIEW [27]. Evolutionary conservation for both CEP85 and STIL was computed using multiple sequence alignments containing 136 non-redundant homologous sequences from the same set of species. The position-specific scores were calculated using a Bayesian algorithm[28]. The conservation scores calculated by CONSURF are divided into nine grades and indicate the relative degree of evolutionary conservation at each amino acid position in the given multiple sequence alignment.

Cell Lines

U-2 OS cells were cultured in standard media and conditions, and were purchased from ATCC. U-2 OS T-REx cells were grown in McCoy 5A medium supplemented with 10% FBS, 2mM GlutaMAX, zeocin (100 µg/ml) and blasticidin (3 µg/ml). U-2 OS T-REx cells with Tet-inducible Myc-tagged PLK4 were a kind gift from E. Nigg, and were maintained in 10% FBS, 2mM GlutaMAX and G418 (0.5 mg/mL). hTERT RPE-1 cells expressing FLAG-tagged Cas9 were grown in DMEM/F12 supplemented with 10% FBS, GlutaMAX and sodium bicarbonate, and were a kind gift from Daniel Durocher. All human cell lines were cultured in a humidified 5% CO_2 atmosphere at 37°C. All cell lines were tested without mycoplasma contamination.

Cloning and Plasmids

FLAG-tagged full-length STIL and CEP85 were cloned into pcDNA5/FRT/TO vector backbone (Life Technologies) using a tetracycline-inducible CMV promoter. The CEP85 (Q640A, E644A or Q640A and E644A) and STIL (L64A, R67A or L64A and R67A) mutants were generated using the QuickChange Site-Directed Mutagenesis protocol.

Generation of stable cell lines

The human CEP85 siRNA (CCUAGAGCAGGAAGUGGCUCAAGAA) target sequence was mutated (TCTCGAACAAGAGGTCGCCCAGGAG) and STIL siRNA (GCUCCAAACAGUUUCUGCUGGAAU) target sequence was mutated (ACTGCAGACCGTGTCCGCCGGCAA) using QuikChange Site-Directed Mutagenesis protocol and cloned into pCMV-TO/FRT-FLAG vector. U-2 OS T-REx cells were co-transfected

with pOG44 (Flp-recombinase expression vector) and pCMV-TO/FRT-FLAG plasmid containing the coding sequence for siRNA resistant human CEP85 and STIL. Transfections were carried out with Lipofectamine 2000 (Invitrogen) according to the manufacturer's protocol. At 24 h post-transfection, cells were selected with 200 μg/ml hygromycin B.

CRISPR

Lentiviral CRISPR/Cas9-mediated gene targeting was used to deplete endogenous CEP85 and CEP192. To achieve complete gene knockout, two gRNAs targeting different exons of CEP85 were used, and their targeting sequences are as followed: CEP85 gRNA1 (CCATCTTAGAGCCAGCACAG) and CEP85 gRNA2 (AGCTGGGCCGTGTCTGGTGA). CEP192 gRNA sequence is GTGCTTAATCCAACTGACCGC. gRNAs were cloned into LentiCRSIPRv2 vector (gift of Feng Zhang, Addgene plasmid #52961). For lentivirus packaging, 293T cells ($1.5x10^6$) were transfected with 3.3 μg LCv2-CEP85 or CEP192 gRNA vector, 2.5μg psPAX2 and 1.7 μg pVSVG using Lipofectamine 3000 (Invitrogen) according to the manufacturer's protocol. 24 h post-transfection, the media was changed with high-serum media containing 30% FBS and virus was collected after 48 h. For lentiviral transduction, 100,000 cells were seeded on 6-well plates and infected with 1ml viral supernatant containing 8 μg/ml polybrene. 24 h post-infection, cells were split and selected in the presence of 10 μg/ml puromycin. Five days post-infection, cells were fixed with ice-cold methanol for immunofluorescence staining, and also collected for western blot experiments.

Immunofluorescence microscopy

Cells were fixed with ice-cold methanol at −20°C for 20 min, and blocked in 1% BSA and 0.05% Tween-20 solution in PBS for one hour. Cells were incubated with the primary antibodies in blocking solution for 2 hours, washed 3x 5min in blocking solution and then incubated with fluorophore-conjugated secondary antibodies (Molecular Probes) and DAPI (0.1 μg/ml) in blocking solution for one hour. After a final wash 3x 5min in blocking solution, cells were inverted and mounted on glass slides with standard mounting solution (ProLong Gold antifade, Molecular Probes). Mouse polyclonal antibody used in this study is anti-CEP85 (H00064793-B01P, dilution 1:200; Abnova). Mouse monoclonal antibodies used in this study are anti-Centrin (clone 20H5, 04-1624, dilution 1:200; Millipore), anti-FLAG (F3165, dilution 1:1000; Sigma), mouse anti-PCNA (PC10, sc-56, dilution 1:200; Santa Cruz) and mouse anti-Pericentrin (ab28144, dilution 1:500; Abcam). Rabbit polyclonal antibodies used in this study are anti-PLK4 pSer305 (dilution 1:500; a kind gift from Tak W. Mak) and anti-CEP192 (A302-324A-1, dilution 1:1000; Bethyl Laboratories) and anti-STIL (dilution 1:1000). This STIL antibody was raised against human, recombinant $STIL^{1-373}$ (containing the N-terminal and C-terminal linker GGS and ENLYFQ respectively) using Davids Biotechnologie GmbH, Regensburg, Germany and was affinity purified from the antiserum using covalently immobilised GST-human $STIL^{1-390}$ (construct as described above) following standard protocols. Goat polyclonal antibodies used in this study are anti-γ-tubulin (sc-7396, dilution 1:200; Santa Cruz), anti-SASS6 (sc-82360, dilution 1:100; Santa Cruz) and anti-C-Myc (ab19234, 1:500; Abcam). Donkey anti-Rabbit Alexa 488/594/647 (Molecular Probes) were used in this study. Cells were imaged on a Deltavision Elite DV imaging system equipped with a sCMOS 2048x2048 pixels2 camera (GE Healthcare). Z stacks (0.2 μm apart) were used, and images were

further deconvolved and maximum intensity projected using softWoRx (v6.0, Applied Precision).

Super resolution microscopy

Super resolution microscopy was essentially performed following standard procedures[29, 30]. Briefly, cells were imaged on a three-dimensional (3D) structured illumination microscope (OMX Blaze v4, GE Biosciences PA) equipped with 405, 445, 488, 514, 568 and 642 nm diode lasers, 4 high-speed sCMOS cameras (scientific CMOS, 2560*2560 pixels, manufactured by PCO), and a ×60/1.42 NA planApochromat oil-immersion objective (Olympus). Multi-channel 3D-SIM image Z stacks were reconstructed, 3D aligned using calibrations based on a GE reference slide and 100 nm-diameter TetraSpeck Microspheres and maximum intensity projected using the softWoRx 6.0 software package (GE). The 3D-SIM modality of our Optical Microscopy eXperiment (OMX) provides an axial resolution of 340-380 nm depending on the imaging wavelength, thus sampling axially every 125nm satisfied the Nyquist criterion for oversampling (GE Biosciences PA).

Image Analysis

Image quantifications were performed in MATLAB. For widefield quantifications, Z stacks were deconvolved and maximum intensity projected using the softWoRx (v6.0, Applied Precision). For 3D-SIM quantifications, we used reconstructed, maximum intensity projected, and aligned images. For each image set, the centrosome was detected as the composite region with the greatest integrated intensity across all channels. Within the composite region, we generated masks (using constant signal-to-noise and size thresholds) for each image channel

specific for the labelled protein. Recruitment to the centrosome for a given label was quantified as the total pixel intensity of its masked region.

Statistical methods

Two-tailed unpaired student *t*-tests were performed for all *p*-values. Individual *p*-values, experiment sample numbers and the number of replicates for statistical testing were indicated in corresponding figure legends. Unless otherwise mentioned, all error bars are S.D, and the asterisk placeholders for *p*-values are $**p<0.01$ and $*p<0.05$.

Western Blots

Cells were collected, lysed in Laemmli sample buffer and digested with benzonase nuclease. Proteins were loaded to 8% SDS-PAGE gel for electrophoresis and transferred to a PVDF membrane (Immobilon-P, Millipore). Membranes were incubated with primary antibodies in TBST (TBS, 0.1% Tween-20) in 5% skim milk powder (BioShop). Blots were washed 3x10 mins in TBST, then incubated with secondary antibodies conjugated to HRP. Western blots were developed using SuperSignal reagents (Thermo Scientific). Mouse polyclonal antibody used in this study is anti-CEP85 (H00064793-B01P, dilution 1:500; Abnova). Mouse monoclonal antibody used in this study are anti-α-tubulin (Clone DM1A, T6199, dilution 1:15000; Sigma-Aldrich), anti-FLAG (F3165, dilution 1:1000; Sigma) and anti-Cyclin A (BF683, dilution 1:1000; Cell Signaling). Rabbit polyclonal antibodies used in this study are anti-CEP192 (A302-324A-1, dilution 1:1000; Bethyl Laboratories) and anti-STIL (A302-441A, dilution 1:500; Bethyl Laboratories). Goat polyclonal antibody used in this study is anti-C-Myc (ab19234, dilution 1:500; Abcam). Uncropped scans of the corresponding Western blots are shown in

Supplementary Figure 11-13. We note that in some cases, the Western blot membranes were cut before probing with specific antibodies to allow the detection of multiple proteins per membrane (Figure 1C and F, Figure 2D and 2I, Figure 6D and 6J, Figure 7C, Supplementary Figure 1e, and Supplementary Figure 9c and 9f).

BioID, mass spectrometry and analysis

BioID and mass spectrometry was performed as described [29]. The network in Supplementary Figure 1a was constructed from SAINT data were imported into Cytoscape 3.2.1 [31], and with the Edge-Weighted Spring Embedded Layout. SAINT analysis of high confidence interactors to generate Supplementary Dataset 1 used the following parameters : TPP >0.9, unique peptides≥2, FDR ~1% [32]. For each prey, peptide counts in each bait sample (two technical replicates of two biological replicates) were compared to those from 14 control runs (see Supplementary Dataset 1).

Supplementary References

1. Taherbhoy, Asad M. *et al.* Atg8 transfer from Atg7 to Atg3: a distinctive E1-E2 architecture and mechanism in the autophagy pathway. *Molecular Cell* **44**, 451-461 (2011).
2. des Georges, A. *et al.* Structure of mammalian eIF3 in the context of the 43S preinitiation complex. *Nature* **525**, 491-495 (2015).
3. Cottee, M.A. *et al.* Crystal structures of the CPAP/STIL complex reveal its role in centriole assembly and human microcephaly. *Elife* **2**, e01071 (2013).
4. van Breugel, M., Wilcken, R., McLaughlin, S.H., Rutherford, T.J. & Johnson, C.M. Structure of the SAS-6 cartwheel hub from Leishmania major. *Elife* **3**, e01812 (2014).
5. Ohashi, Y. *et al.* Characterization of Atg38 and NRBF2, a fifth subunit of the autophagic Vps34/PIK3C3 complex. *Autophagy* **12**, 2129-2144 (2016).
6. Leslie, A.G.W. & Powell, H.R. Processing Diffraction Data with Mosflm. *Evolving Methods for Macromolecular Crystallography* **245**, 41-51 (2007).
7. Kabsch, W. XDS. *Acta Crystallogr D Biol Crystallogr* **66**, 125-132 (2010).
8. Evans, P. Scaling and assessment of data quality. *Acta Crystallogr D Biol Crystallogr* **62**, 72-82 (2006).
9. Evans, P.R. & Murshudov, G.N. How good are my data and what is the resolution? *Acta Crystallogr D Biol Crystallogr* **69**, 1204-1214 (2013).
10. Ness, S.R., de Graaff, R.A., Abrahams, J.P. & Pannu, N.S. CRANK: new methods for automated macromolecular crystal structure solution. *Structure* **12**, 1753-1761 (2004).
11. McCoy, A.J. *et al.* Phaser crystallographic software. *J Appl Crystallogr* **40**, 658-674 (2007).
12. Cowtan, K. The Buccaneer software for automated model building. 1. Tracing protein chains. *Acta Crystallogr D Biol Crystallogr* **62**, 1002-1011 (2006).
13. Emsley, P. & Cowtan, K. Coot: model-building tools for molecular graphics. *Acta Crystallogr D Biol Crystallogr* **60**, 2126-2132 (2004).
14. Afonine, P.V. *et al.* Towards automated crystallographic structure refinement with phenix.refine. *Acta Crystallogr D Biol Crystallogr* **68**, 352-367 (2012).
15. Sheldrick, G.M. A short history of SHELX. *Acta Crystallogr A* **64**, 112-122 (2008).
16. Winn, M.D. *et al.* Overview of the CCP4 suite and current developments. *Acta Crystallogr D Biol Crystallogr* **67**, 235-242 (2011).
17. Murshudov, G.N. *et al.* REFMAC5 for the refinement of macromolecular crystal structures. *Acta Crystallogr D Biol Crystallogr* **67**, 355-367 (2011).
18. Al-Jassar, C. *et al.* The Ciliopathy-Associated Cep104 Protein Interacts with Tubulin and Nek1 Kinase. *Structure* **25**, 146-156 (2017).
19. Marty, M.T. *et al.* Bayesian deconvolution of mass and ion mobility spectra: from binary interactions to polydisperse ensembles. *Anal Chem* **87**, 4370-4376 (2015).
20. Schmidt, C. & Robinson, C.V. A comparative cross-linking strategy to probe conformational changes in protein complexes. *Nature protocols* **9**, 2224 (2014).
21. Shevchenko, A., Tomas, H., Havli, J., Olsen, J.V. & Mann, M. In-gel digestion for mass spectrometric characterization of proteins and proteomes. *Nature protocols* **1**, 2856 (2007).
22. Schmidt, C. *et al.* Comparative cross-linking and mass spectrometry of an intact F-type ATPase suggest a role for phosphorylation. *Nature Communications* **4**, 1985 (2013).
23. Schuck, P. On the analysis of protein self-association by sedimentation velocity analytical ultracentrifugation. *Anal Biochem* **320**, 104-124 (2003).
24. Altschul, S.F. *et al.* Gapped BLAST and PSI-BLAST: a new generation of protein database search programs. *Nucleic Acids Research* **25**, 3389-3402 (1997).

25. Ashkenazy, H. *et al.* ConSurf 2016: an improved methodology to estimate and visualize evolutionary conservation in macromolecules. *Nucleic Acids Research* **44**, W344-W350 (2016).
26. Katoh, K. & Standley, D.M. MAFFT Multiple Sequence Alignment Software Version 7: Improvements in Performance and Usability. *Molecular Biology and Evolution* **30**, 772-780 (2013).
27. Waterhouse, A.M., Procter, J.B., Martin, D.M.A., Clamp, M. & Barton, G.J. Jalview Version 2—a multiple sequence alignment editor and analysis workbench. *Bioinformatics* **25**, 1189-1191 (2009).
28. Mayrose, I., Graur, D., Ben-Tal, N. & Pupko, T. Comparison of Site-Specific Rate-Inference Methods for Protein Sequences: Empirical Bayesian Methods Are Superior. *Molecular Biology and Evolution* **21**, 1781-1791 (2004).
29. Gupta, Gagan D. *et al.* A Dynamic Protein Interaction Landscape of the Human Centrosome-Cilium Interface. *Cell* **163**, 1484-1499 (2015).
30. Comartin, D. *et al.* CEP120 and SPICE1 cooperate with CPAP in centriole elongation. *Current Biology* **23**, 1360-1366 (2013).
31. Shannon, P. *et al.* Cytoscape: A Software Environment for Integrated Models of Biomolecular Interaction Networks. *Genome Research* **13**, 2498-2504 (2003).
32. Choi, H. *et al.* SAINT: probabilistic scoring of affinity purification-mass spectrometry data. *Nat Meth* **8**, 70-73 (2011).
