# Supplementary material for: Direct binding of CEP85 to STIL ensures robust PLK4 activation and efficient centriole assembly
Source: Nat Commun. 2018 Apr 30;9:1731. doi: 10.1038/s41467-018-04122-x (PMC5928214; doi:10.1038/s41467-018-04122-x)
Supplement: Supplementary file 4 — Description of Additional Supplementary Files [file 41467_2018_4122_MOESM4_ESM.pdf]

**Supplementary Information**

# Direct binding of CEP85 to STIL ensures robust PLK4 activation and efficient centriole assembly

**Liu et al.**

Description of Additional Supplementary Files

File Name: Supplementary Data 1

Description: High confidence proximity interactors for centriole duplication factors.

## Supplementary Data 1 legend

**Supplementary Data 1.** (**a**) High confidence proximity interactors for centriole duplication factors (TPP > 0.9, unique peptides ≥ 2, FDR ~1%). Font and background colours as described in Table inset. SAINT analysis conducted using 14 control runs (FLAG-BirA* alone). For each Bait protein, two technical replicates were conducted on each of two biological samples (shown only for *bona fide* interactors with a FDR~1%); (**b**, **c**) Mass spectrometry raw analysis (TPP>0.9, unique peptides ≥ 2); (**d**) Bait and interactor annotations (see Supplementary Figure 1a); (**e**) Cilia and Centrosome data base (CCDB), as assembled in Gupta *et al* [1](Supplementary Table 3 in that reference).

## Supplementary Reference

1. Gupta, Gagan D. *et al.* A Dynamic Protein Interaction Landscape of the Human Centrosome-Cilium Interface. *Cell* **163**, 1484-1499 (2015).
