# Supplementary material for: Author Correction: Direct binding of CEP85 to STIL ensures robust PLK4 activation and efficient centriole assembly
Source: Nat Commun. 2026 Nov 13;17:11674. doi: 10.1038/s41467-026-79190-5 (PMC13768342; doi:10.1038/s41467-026-79190-5)

Figure 2

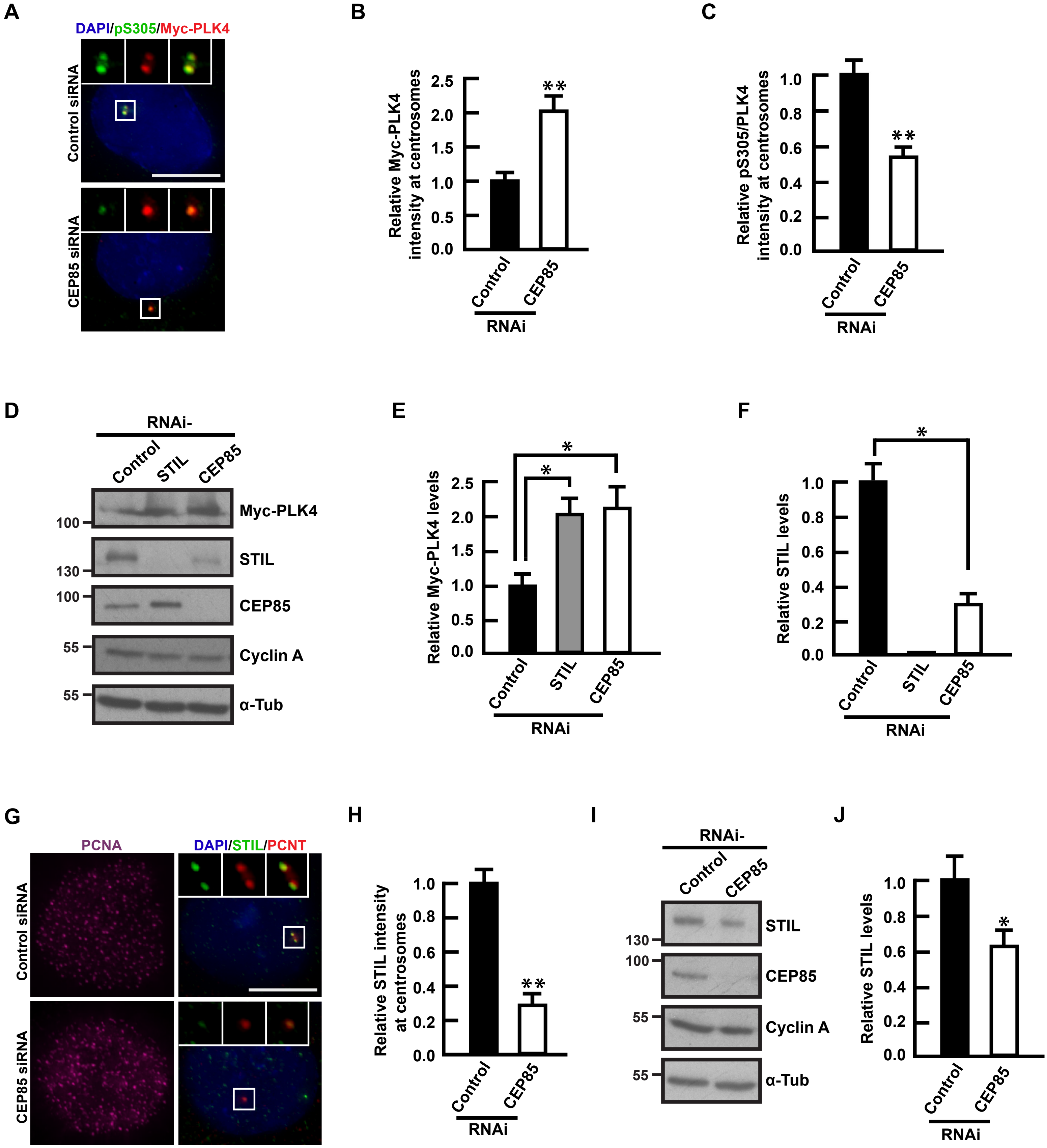

Liu et al. 2018

Supplementary Figure 11

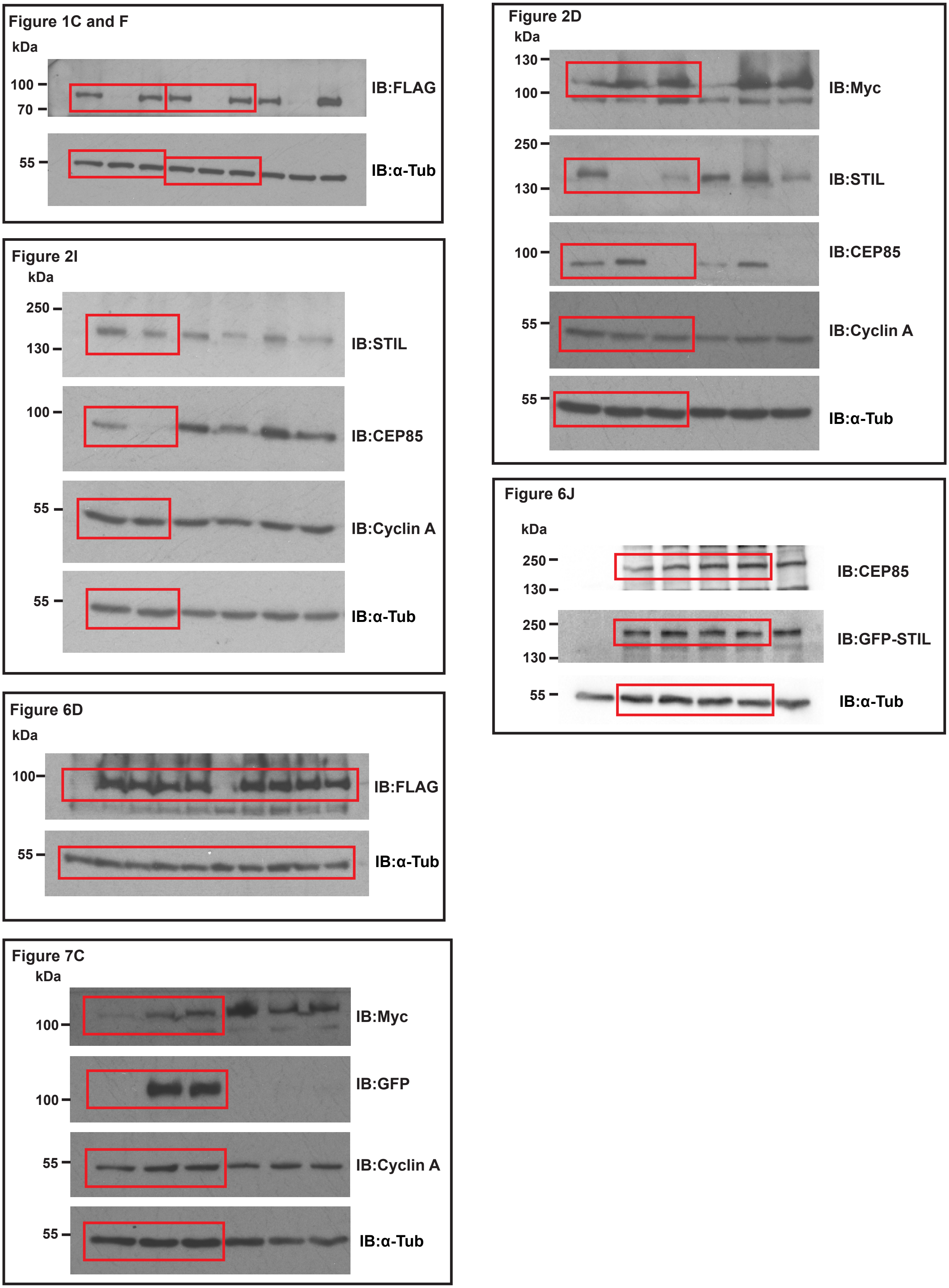

Supplement: Supplementary file 1 [file 41467_2026_79190_MOESM1_ESM.pdf]
